# Overexpression of adhesion molecules and barrier molecules is associated with differential infiltration of immune cells in non-small cell lung cancer

**DOI:** 10.1038/s41598-018-19454-3

**Published:** 2018-01-18

**Authors:** Young Kwang Chae, Wooyoung M. Choi, William H. Bae, Jonathan Anker, Andrew A. Davis, Sarita Agte, Wade T. Iams, Marcelo Cruz, Maria Matsangou, Francis J. Giles

**Affiliations:** 1 Robert H. Lurie Comprehensive Cancer Center of Northwestern University, Chicago, 60611 USA; 2 Northwestern University Feinberg School of Medicine, Chicago, 60611 USA

## Abstract

Immunotherapy is emerging as a promising option for lung cancer treatment. Various endothelial adhesion molecules, such as integrin and selectin, as well as various cellular barrier molecules such as desmosome and tight junctions, regulate T-cell infiltration in the tumor microenvironment. However, little is known regarding how these molecules affect immune cells in patients with lung cancer. We demonstrated for the first time that overexpression of endothelial adhesion molecules and cellular barrier molecule genes was linked to differential infiltration of particular immune cells in non-small cell lung cancer. Overexpression of endothelial adhesion molecule genes is associated with significantly lower infiltration of activated CD4 and CD8 T-cells, but higher infiltration of activated B-cells and regulatory T-cells. In contrast, overexpression of desmosome genes was correlated with significantly higher infiltration of activated CD4 and CD8 T-cells, but lower infiltration of activated B-cells and regulatory T-cells in lung adenocarcinoma. This inverse relation of immune cells aligns with previous studies of tumor-infiltrating B-cells inhibiting T-cell activation. Although overexpression of endothelial adhesion molecule or cellular barrier molecule genes alone was not predictive of overall survival in our sample, these genetic signatures may serve as biomarkers of immune exclusion, or resistance to T-cell mediated immunotherapy.

## Introduction

Immunotherapy has emerged as a promising option for treating lung cancer. Compared to conventional chemotherapy, which focuses on inhibiting the process of cell division, immunotherapy aims to modulate the patient’s anti-tumor immune response, resulting in favorable survival outcome and fewer side effects^1^. While increased cytotoxic T-cell infiltration into the tumor microenvironment (TME) is associated with improved clinical outcomes^2^, the mechanisms controlling this infiltration are still being studied. Previous research has established the roles of chemokine receptors, T-cell homing receptors^3^, and antigen presentation that interfere with T-cells in the TME^4^. However, the significance of how endothelial adhesion molecules (EAMs) and cellular barrier molecules (CBMs) mechanistically interact with immune cells in lung cancer is poorly understood.

**Figure 1:**
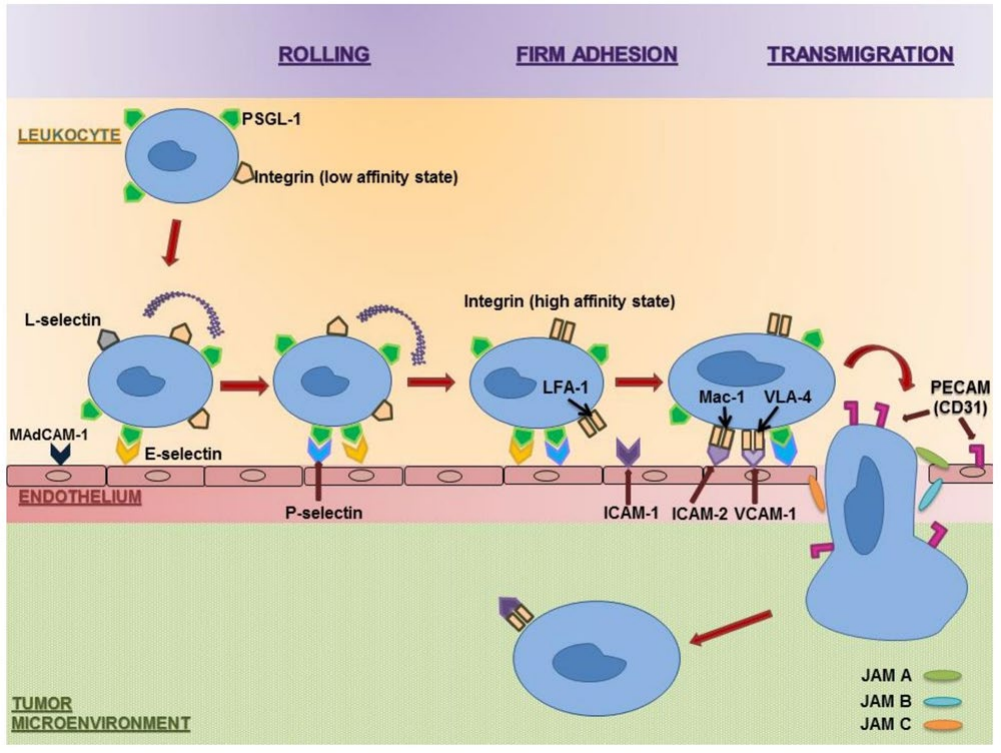
Schematic model of leukocyte adhesion cascade involved in immune cell infiltration. A step by step process involving rolling, firm adhesion, and transmigration moves the leukocyte in the circulatory system towards the tumor site. This cascade is mediated by various molecules interacting with each other as above. Abbreviations: PSGL-1: P-selectin glycoprotein ligand-1, MAdCAM-1: Mucosal vascular addressin cell adhesion molecule 1, LFA-1: Lymphocyte function-associated antigen 1, Mac-1: Macrophage-1 antigen, VLA-4: Very Late Antigen-4, ICAM-1/2: Intercellular adhesion molecule 1/2, VCAM-1: Vascular cell adhesion molecule-1, PECAM: Platelet Endothelial Cell Adhesion Molecule, JAM A/B/C: Junctional adhesion molecule A/B/C.

The endothelium separates circulating immune cells and the TME. It is necessary for immune cells to penetrate this barrier to reach the tumor. This requires cell-cell interactions via adhesion molecules such as selectin and integrin. Immune cells first undergo “rolling,” which is initiated by interactions between endothelial P/E-selectins, PNAd, and MAdCAM-1, as well as leukocyte L-selectin, PSGL-1, and E-selectin ligand^5^ (Fig. 1). This step is reversible unless firm adhesion occurs. Firm adhesion is mediated by the interaction of endothelial ICAM-1/2, VCAM-1, MAdCAM-1 with leukocyte α4β7 integrin, α4β1 integrin (VLA4), and αLβ2 integrin (LFA-1). Previous studies on monocytes and neutrophils have demonstrated that αMβ2 integrin (Mac-1) triggers apical leukocyte flattening and crawling along the endothelium^6^. Transmigration is the final stepwhich is regulated by endothelial PECAM-1 and JAM-A/B/C, interacting with leukocyte PECAM-1, LFA-1, VLA-4, and Mac-1.

For immune cells to interact with tumor cells, they must also traverse cell-cell junctions, including tight junction, adherens junction, and desmosomes. These protein-rich regions form molecular barriers between the TME and circulating immune cells. Some sites such as the retina, brain, and testes, are immunologically privileged due to the presence of immunosuppressive signaling and a physical barrier (comprised of cell-cell junctions) at the blood-tissue interface^7–9^. As T-cells cannot penetrate these sites, the risk of an autoimmune reaction is reduced. At the blood-brain and blood-retina barriers, tight junctions prevent direct contact between the blood and tissue. While at the blood-testis barrier, both tight junctions and desmosomal adhesions prevent direct contact between the blood and tissue^10^. Desmosomes are adhesion junctions composed of various proteins, including cadherins, which are anchored to intermediate filaments by desmoplakins. In lung and colon cancer, elevated levels of cadherin and desmocollin 3 (DSC3), were associated with improved prognosis. However, in melanoma, elevated DSC3 was linked to increased metastatic risk^11–13^.

Elevated expression of EAM and CBM genes are expected to facilitate and impede immune cell infiltration, respectively. Previous studies reported a correlation between EAM gene expression and increased CD8 T-cell infiltration in merkel cell carcinoma, pancreatic carcinoma, and hepatocellular carcinoma^14,15^. Inversely, overexpression of CBM genes were shown to be associated with decreased CD8 T-cell infiltration in human melanoma and ovarian carcinoma^10^. However, these connections have not yet been verified in the context of human lung cancer. Here, we report results that contradict prior hypotheses regarding the relationship between immune cell infiltration and the expression levels of EAM and CBM genes.

## Methods

Gene expression data were obtained from The Cancer Genome Atlas (TCGA) project and analyzed through cbioportal.org^16,17^. These data contain mRNA-seq gene expression data from 522 patients with lung adenocarcinoma and 504 patients with lung squamous cell carcinoma (SCC). The mRNA z-scores of 812 immune metagene signatures from previous publications were evaluated using the Gene Set Enrichment Analysis (GSEA) from the Broad Institute^18^. Any immune cell types with a false discovery rate (q-value) lower than or equal to 10% were considered as positively infiltrating, and a total of 31 distinct immune cells were analyzed for each tumor samples^19^.

Overall immune cell infiltration landscape was analyzed by Microsoft Excel and Graphpad Prism version 7.03. Expression of EAM genes and CBM genes^10^ in Tables 1 and 2 were assessed. EAM genes were divided into 3 classifications: rolling, firm adhesion, and transmigration. As for all 3 EAM genes, a total of 515 and 501 patient samples were sequenced for lung adenocarcinoma and SCC, respectively. CBM genes were divided into 3 types: tight junction, adherens junction, and desmosome. 515 patient samples for all 3 CBM gene types were sequenced for lung adenocarcinoma. 177 tight junction/adherens junction patient samples and 501 desmosome patient samples were sequenced for lung SCC.

**Table 1:** List of endothelial adhesion molecules and related genes.

**Endothelial Adhesion Molecule genes**	
Rolling	*SELP* (P-selectin) *SELE* (E-selectin) *MADCAM1* (mucosal vascular addressin cell adhesion molecule 1) *NTAN1* (peripheral node addressin) *SELPLG* (P selectin glycoprotein ligand 1) *GLG1* (E selectin ligand 1) *SELL* (L-selectin).
Firm adhesion	*ICAM1/2* (intercellular adhesion molecule 1/2) *VCAM1* (vascular cell adhesion molecule) *MADCAM1* (mucosal vascular addressin cell adhesion molecule 1) *ITGA4/AM/AL* (α4/αM/αL integrin) *ITGB1/B2/B7* (β1/β2/β7 integrin)
Transmigration	*JAM1/2/3* (junctional adhesion molecule 1/2/3) *ITGA4/AM/AL* (α4/αM/αL integrin) *ITGB1/B2* (β1/β2 integrin)

**Table 2:** List of cellular barrier molecules and related genes.

**Cellular Barrier Molecule genes**	
Tight junction	*CLDN1/5/7* (claudin-1/5/7) *JAM1/2* (junctional adhesion molecule 1/2) *TJP1/2* (zona occluden 1/2)
Adherens junction	*CDH1/5* (E/VE-cadherin) *CTNNA1/B1* (α/β-catenin) *CTNND1* (p120 catenin)
Desmosome	*DSC3* (desmocollin 3) *PKP3* (plakophilin 3) *JUP* (junction plakoglobin) *DSP* (desmoplakin) *DST* (dystonin; bullous pemphigoid antigen 1) *PPL* (periplakin) *DSG1* (desmoglein 1)

Overexpression was defined as mRNA z-score >2.0. P values were statistically derived from Fisher’s exact and Chi squared tests via the analysis function of Prism. Significance was assumed when p value was <0.05. Overall patient survival was assessed through the cBio Cancer Genomics Portal and Prism using Kaplan-Meier curves. Tendency towards co-occurence between two genes was derived from the Bioportal which calculated an odds ratio (OR) for each pair of query genes (G1, G2). OR = (number of cases altered in both genes ∗ number of cases altered in neither genes)/(number of cases altered in G1 but not G2 ∗ number of cases altered in G2 but not G1)^16^.

## Results

### Endothelial adhesion molecule overexpression is associated with lower CD4 and CD8 T-cell, and higher activated B-cell and regulatory T-cell infiltration in human lung adenocarcinoma and squamous cell carcinoma

**Figure 2:**
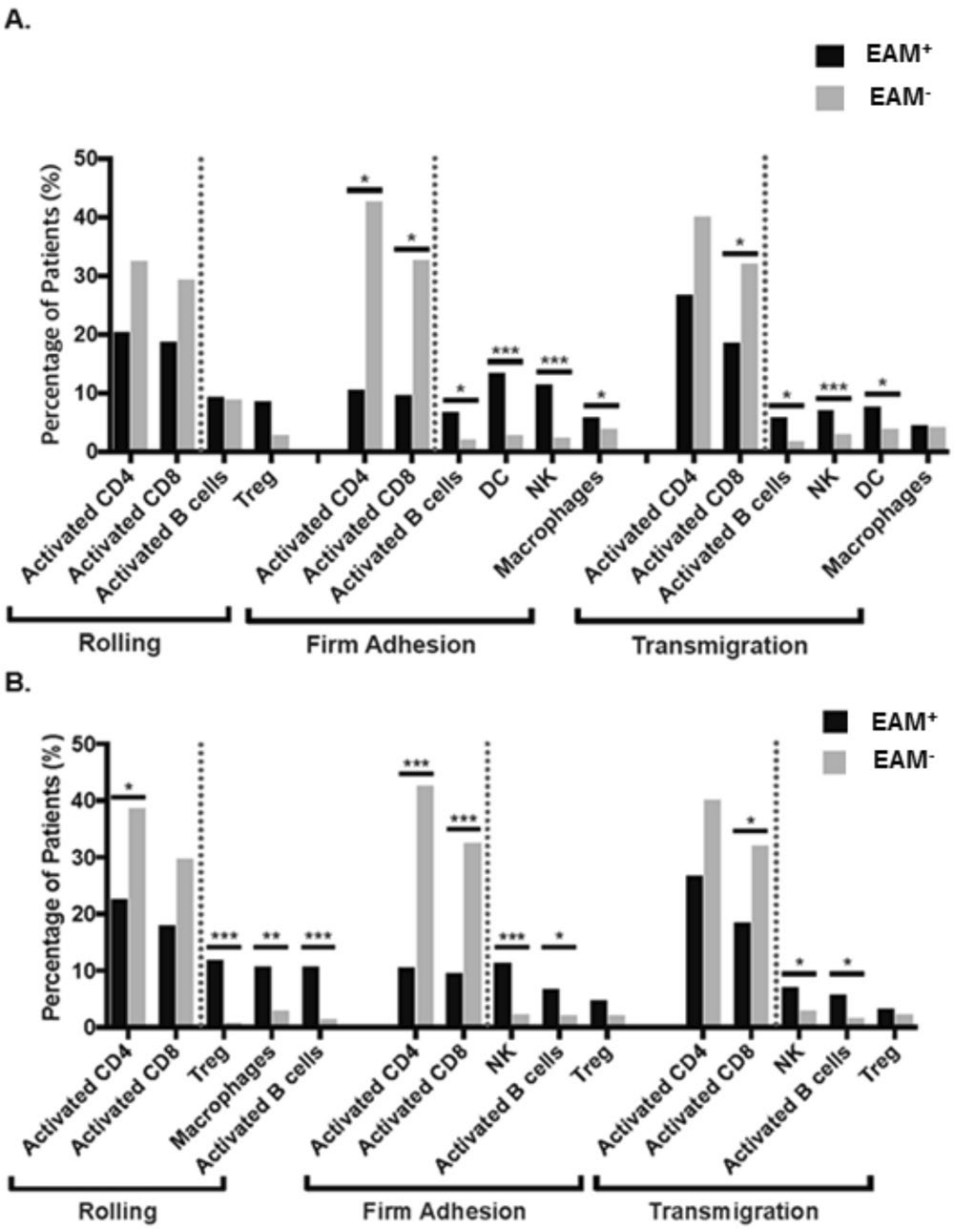
Immune cell infiltration landscape by EAM gene status. (**A**) In lung adenocarcinoma, overexpression of EAM genes was associated with lower infiltration of activated CD4/CD8 T-cells, but higher infiltration of activated B-cells. (**B**) In lung SCC, overexpression of EAM genes was associated with lower infiltration of activated CD4/CD8 T-cells, but higher infiltration of activated B-cells/Treg cells. *p < 0.05, **p < 0.01, ***p < 0.001, Abbreviations: EAM+ overexpression group: EAM-, Remaining group: Treg, regulatory T-cells: NK, natural killer cells, DC, dendritic cells.

In lung adenocarcinoma, 22.9%, 26%, and 31.8% of tumor samples displayed overexpression of rolling genes, firm adhesion genes, and transmigration genes, respectively. Interestingly, samples with rolling gene overexpression contained a trending lower infiltration rate of activated CD4 and CD8 T-cells and higher infiltration rate of regulatory T-cells (Tregs) (Fig. 2A). In firm adhesion overexpressing samples, there was significantly decreased infiltration of activated CD4 T-cells and activated CD8 T-cells, and significantly increased infiltration of activated B-cells, macrophages, dendritic cells, and natural killer cells. Furthermore, samples overexpressing transmigration genes displayed significantly decreased intra-tumoral activated CD8 T-cells with significantly increased infiltration of activated B-cells, dendritic cells, and natural killer cells. The overall immune cell landscapes in lung adenocarcinoma in samples with or without EAM overexpression are presented in Figure S1.

In addition, we analyzed the impact of EAM gene expression on immune cell infiltration in lung SCC. Similar to lung adenocarcinoma, 16.7% of samples overexpressed rolling genes, 20.9% overexpressed firm adhesion genes, and 31.3% overexpressed transmigration genes. In the rolling overexpression group, we observed significantly decreased infiltration of activated CD4 T-cells and increased infiltration of macrophages, activated B-cells, and regulatory T-cells (Fig. 2B). In the firm adhesion overexpression group, infiltration of activated CD4 and CD8 T-cells were significantly lower, whereas infiltration of activated B-cells, dendritic cells, and natural killer cells were higher. Finally, samples overexpressing transmigration genes displayed significantly decreased infiltration of activated CD8 T-cells, and significantly increased infiltration of activated B-cells, and natural killer cells. The overall immune cell landscapes of lung SCC in the setting of EAM gene overexpression are shown in Figure S2. Therefore, in both lung adenocarcinoma and SCC, overexpression of EAM genes at all steps of adhesion was strongly linked to decreased infiltration of activated CD8 and CD4 lymphocytes, and increased infiltration of Tregs, macrophages, activated B-cells, and NK cells.

### Cellular barrier molecule overexpression correlates with higher activated CD4 and CD8 T-cell, and lower activated B-cell and regulatory T-cell infiltration in human lung adenocarcinoma

**Figure 3:**
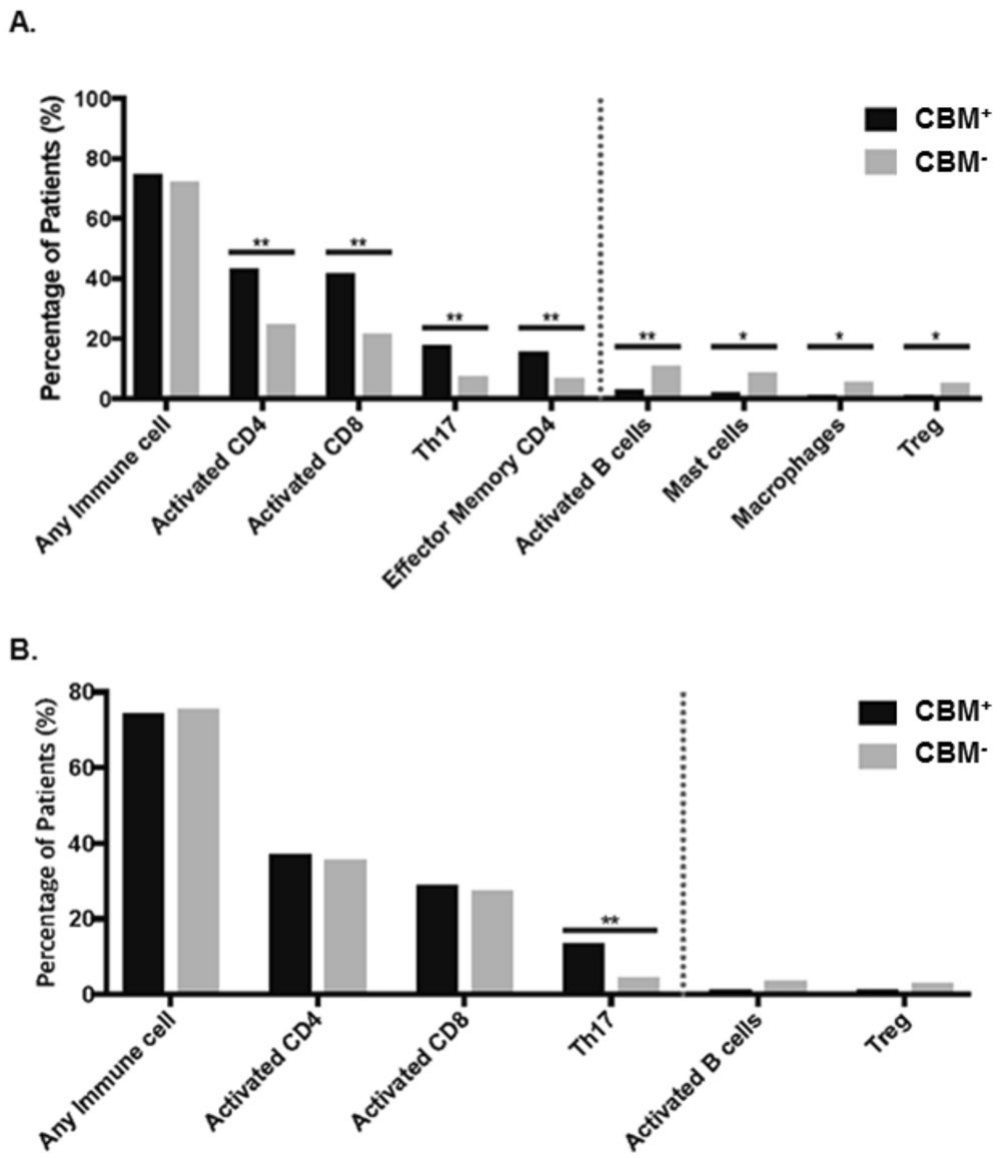
Immune cell infiltration landscape by desmosome gene status. (**A**) In lung adenocarcinoma, overexpression of desmosome genes was associated with higher infiltration of activated CD4/CD8/effector memory CD4 T-cells and Th17 cells, but lower infiltration of activated B/Treg/mast cells and macrophages. (**B**) In lung SCC, overexpression of desmosome genes was associated with higher infiltration Th17 cells. *p < *0.05*, **p < *0.01*, *Abbreviations: CBM*^+^, *overexpression group: CBM*^−^, *Remaining group*.

In lung adenocarcinoma, 30.0% of samples overexpressed tight junction genes, 18.4% overexpressed adherens junction genes, and 27.4% overexpressed desmosome genes. Upon analysis of the tumor immunophenotype, samples overexpressing both tight junction and adherens junction genes did not show any alterations of immune cell infiltration (Figure S3). On the contrary, samples containing overexpression of desmosome genes also showed significantly increased infiltration of activated CD4, activated CD8, effector memory CD4 T-cells, and Th17 cells, with decreased infiltration of activated B-cells, mast cells, macrophages, and Tregs (Fig. 3A). The overall immune cell landscapes in the three distinct barrier molecule types in lung adenocarcinoma are shown in Figure S3.

Upon analysis of lung SCC, 49.7% of samples overexpressed tight junction genes, 11.3% overexpressed adherens junction genes, and 25.1% overexpressed desmosome genes. Again, there were no significant differences in the immune cell infiltration of samples containing tight junction or adherens junction gene overexpression. However, in those overexpressing desmosome genes, there was increased infiltration of Th17 cells and decreased infiltration of activated B-cells (Fig. 3B). The total immune cell landscapes in the three distinct barrier molecule types in lung SCC are presented in Figure S4. Overall, consistent in both lung cancer histologies, increased desmosome gene expression was associated with increased Th17 anti-tumor infiltration.

### Endothelial adhesion molecule and desmosome expression status are independent of each other

**Figure 4:**
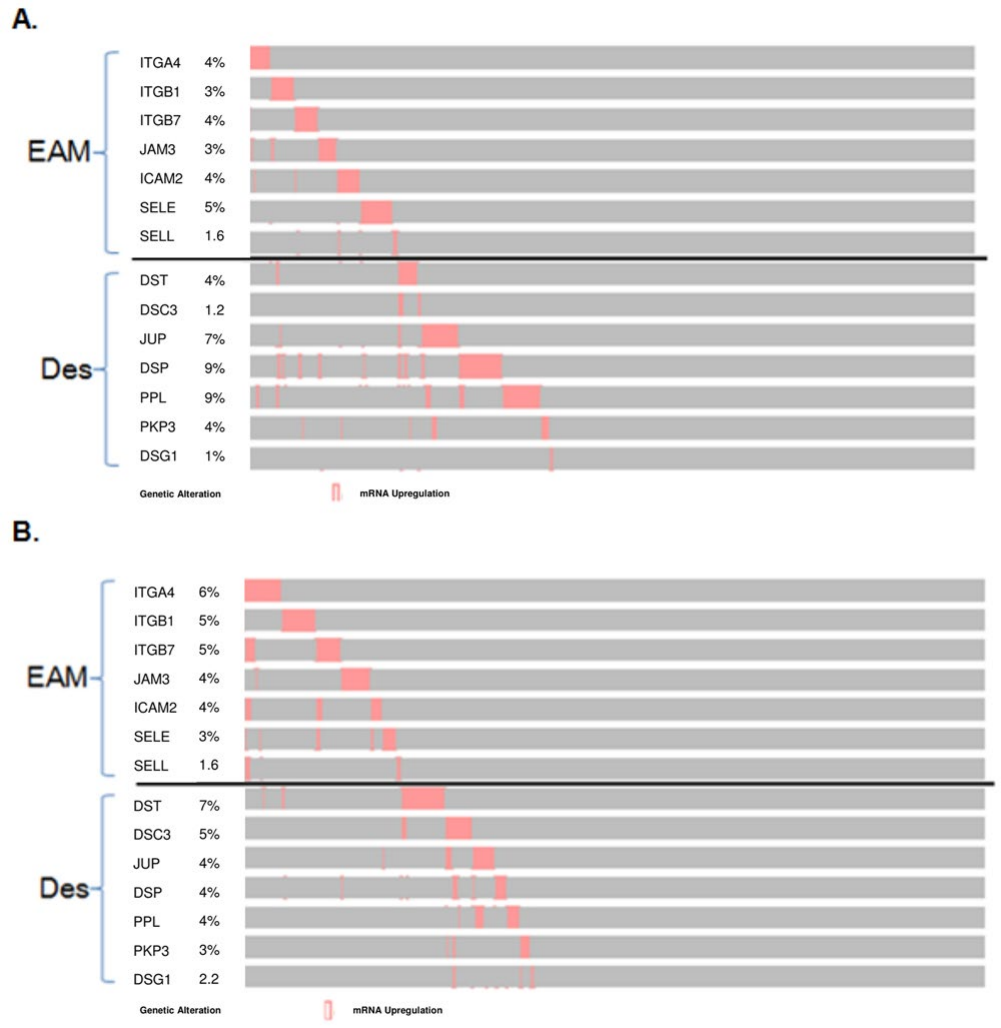
OncoPrint of EAM and desmosome genes. Overexpression of genes in each tumor sample is shown by a red bar (z score > 2.0). EAM genes are grouped at the top, and desmosome genes are grouped at the bottom. The image was obtained from the TCGA database from cbioportal.org. (**A**) Lung adenocarcinoma. (**B**) Lung SCC. *Abbreviations: EAM, endothelial adhesion molecule: Des, desmosome*.

The distribution pattern of upregulated EAM genes and desmosome genes were compared using OncoPrint (cbioportal.org), a compact mean of visualizing distinct mRNA expression changes across a set of cases^16,17^. In both lung adenocarcinoma and SCC, the distribution pattern of EAM and desmosome gene overexpressing samples was independent of each other (Fig. 4). To better understand the association between these two set of genes statistically, mutual exclusivity of each gene was analyzed using p values determined by a Fisher’s exact test based on cbioportal.org. In lung adenocarcinoma, only the EAM gene ITGB1 and the desmosome gene DST significantly converged towards co-occurrence (p value = 0.021) (Table S1). On the contrary, no genes between the EAM and desmosome groups significantly co-occurred in lung SCC.

### Elevated CD8 T-cell signature gene expression is associated with elevated endothelial adhesion molecule expression

**Figure 5:**
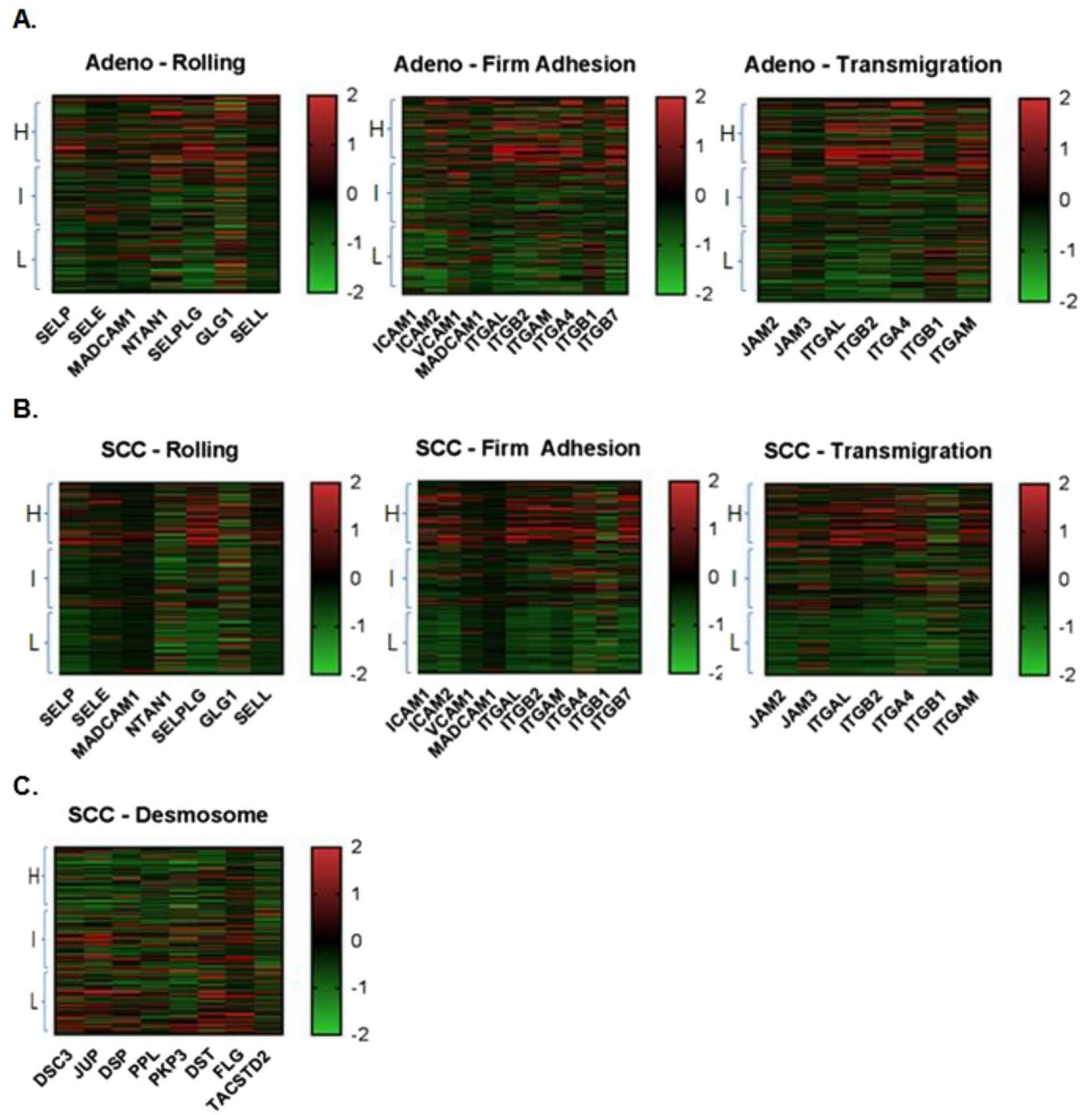
Heat maps of CD8 T-cell signature gene expression in each EAM and desmosome gene status. (**A**) In lung adenocarcinoma, elevated expression of EAM genes was clustered in high CD8 T-cell score group. (**B**) In lung SCC, elevated expression of EAM genes was also clustered in high CD8 T-cell score group. (**C**) In lung SCC, elevated expression of desmosome genes was clustered in low CD8 T-cell score group. *Right column bars indicate mRNA-seq z scores. Abbreviations: H, high: I, intermediate: L, low expression of CD8 T-cell signature genes*.

The CD8 T-cell score was generated from the mean gene expression z score of four CD8 T-cell signature genes (CD8A, CD8B, IFNG, and PRF1)^20^. The CD8 T-cell scores were divided into three groups: high (highest one-third), intermediate (middle one-third), and low (lowest one-third). 171 patient samples were included in each group with lung adenocarcinoma and 167 patient samples were included in each group with lung SCC. In both lung adenocarcinoma and SCC, the samples overexpressing EAM genes were clustered in the high CD8 T-cell score group (Fig. 5). Upon analysis of CBM genes, samples overexpressing tight junction and adherens junction genes did not correlate with CD8 T-cell score (Figure S5). However, lung SCC samples overexpressing desmosome genes correlated with low CD8 T-cell score (Fig. 5). Therefore, increased expression of genes of all stages of immune cell-endothelial adhesion were linked to high CD8 T-cell score and functionality, while increased expression of desmosome genes were linked to lower CD8 T-cell score.

### Endothelial adhesion molecule overexpression is independent of β-catenin or transforming growth factor-β (TGF-β) overexpression

Among many pathways involved in immune modulation, β-catenin, TGF-β, and INF-γ pathways clearly play important roles in modulating immune cell infiltration. Active β-catenin signaling inhibits infiltration of CD8 T-cells in tumor tissue by inhibiting CD103 dendritic cells^21–23^. Additionally, TGF-β signaling is an effective inducer of apoptosis in CD8 T-cells^24–26^. In contrast, INF-γ signaling contributes to CD8 T-cell recognition and PD-L1 blockade^27^. We compared seven EAM genes (*ITGA4, ITGB1, ITGB7, JAM3, ICAM2, SELE, SELL*) and seven CBM genes (*DSC3, PKP3, JUP, DSP, DST, PPL, DSG1*) with six β-catenin pathway genes (*EFNB3, HNF1A, MYC, VEGFA, CCND1, JUN*), six TGF-β pathway genes (*TGFB1, TGFB2, SMAD2, SMAD7, SMAD9, CLIC4*), and six INF- γ pathway genes (*IFNG, IFNGR1, IFNGR2, JAK1, JAK2, STAT1*) to identify any potential relationship.

Overexpression of EAM/CBM genes and β-catenin/TGF-β/INF-γ pathway genes occurred independently of each other in both lung adenocarcinoma and SCC (Figure S6). Upon further analysis of EAM and β-catenin/TGF-β/INF-γ genes respectively, 2 out of 42 gene pairs (JAM3-HNF1A, SELE-JUN), 6 out of 42 gene pairs (*JAM3-SMAD9, ITGB1-TGFB1, ITGB1-TGFB2,ITGB1-SMAD2, ITGB1-CLIC4, ITG4-CLIC4*), and 8 out of 42 gene pairs (*IFNG-ITGA4, IFNG-ITGB7, IFNG-ICAM2, IFNGR1-ICAM2, JAK1-ITGB7, JAK2-ITGA4, JAK2-ITGB7, STAT1-ITGA4*) in lung adenocarcinoma, and 3 out of 42 gene pairs (*JAM3-HNF1A, JAM3-EFNB3, ITGB7-CCND1*), 5 out of 42 gene pairs (*JAM3-SMAD9, ITGB1-TGFB2, ITGB1-SMAD7, ICAM2-SMAD7, SELE-SMAD7*), and 15 out of 42 gene pairs (*STAT1-ITGB7, JAK1-SELL, IFNG-ITGB7, STAT1-ICAM2, JAK1-ITGB7, IFNG-ICAM2, JAK1-SELE, JAK1-ITGA4, JAK2-ITGA4, JAK1-ICAM2, JAK2-ICAM2, IFNGR1-ITGB1, JAK2-ITGB7, JAK2-SELE, JAK2-SELL*) in SCC displayed statistically significant tendency towards co-occurrence (Table S2). Additionally with CBM and β-catenin/TGF-β/INF-γ genes respectively, 4 out of 42 gene pairs (*HNF1A-PKP3, VEGFA-DSP, JUN-DST, HNF1A-JUP*), 2 out of 42 gene pairs (*TGFB2-PPL, CLIC4-DSC3*), and 1 out of 42 gene pairs (*JAK1-DSP*) in lung adenocarcinoma, and 1 out of 42 gene pairs (*VEGFA-DST*), 5 out of 42 gene pairs (*TGFB1-JUP, TGFB1-PKP3, TGFB1-DSC3, SMAD2-DSC3, CLIC4-PKP3*), and none of the 42 gene pairs in SCC displayed statistically significant tendency towards co-occurrence (Table S3).

### Overexpression of endothelial adhesion molecules and cellular barrier molecules is not related to better survival outcome or tumor mutation burden

Finally, we analyzed the impact of EAM and CBM gene overexpression on overall survival and tumor mutation burden. In both lung adenocarcinoma and SCC, there were no significant differences in overall survival with respect to gene expression (Figure S7, S8). Similarly, tumor mutation burden remained unchanged regardless of EAM and CBM expression status in both cancer subtypes, other than the lung adenocarcinoma desmosome overexpression group containing a decreased mutation count (mean difference = 99.11, p value = 0.0277) (Figure S9).

## Discussion

Recent pre-clinical and clinical studies in cancer immunotherapy have drawn attention to the importance of understanding the immunological tumor microenvironment. Specifically, in non-small cell lung cancer (NSCLC), immune checkpoint blockade has provided treatment efficacy by augmenting the cytotoxicity of tumor infiltrating lymphocytes^28,29^. Targeting immune checkpoint molecules, such as cytotoxic T lymphocyte associated protein 4 (CTLA-4) and programmed cell death protein 1 (PD-1), has shown promising results in patients with NSCLC^30,31^. However, only approximately 20% of NSCLC patients respond to PD-L1 therapy^32^, while a large proportion of responders develop drug resistance^33^. The causes of differential responsiveness among these patients still remain largely unknown.

One possible mechanism of controlling T-cell infiltration, and subsequent efficacy with immune checkpoint inhibitors, is by modulation of mechanical barriers created by endothelial or cell-cell junctions. In the present study, we utilized mRNA expression scores of EAM and CBM genes in NSCLC, specifically adenocarcinoma and SCC. EAM genes were divided into 3 groups: rolling, firm adhesion, and transmigration. Rolling overexpression group showed lower infiltration of activated CD4 T-cells, but higher infiltration of activated B-cells, Tregs, and macrophages in lung SCC (Fig. 2). The firm adhesion overexpression group had lower infiltration of activated CD4 and CD8 T-cells, but higher infiltration of activated B-cells and NK cells in both lung adenocarcinoma and SCC. The transmigration overexpression group similarly showed lower infiltration of activated CD8 T-cells, but higher infiltration of activated B-cells and NK cells in both types of lung cancer. Patient samples that had elevated expression of EAM genes were found to be clustered in sample groups with high CD8 T-cell signature scores (Fig. 5), as defined by CD8A, CD8B, IFNG, and PRF1. Yet, overall survival and disease free survival were unchanged with or without EAM gene overexpression (Figure S7).

While we expected that higher expression of EAM genes would be linked to greater immune cell infiltration, our study revealed a reversed relationship regarding activated CD4 and CD8 T-cells. This may be due to the cytokines produced in the TME. Tumors produce soluble factors such as interleukin 10 (IL-10), vascular endothelial growth factor A (VEGF-A), and prostaglandin E2 (PGE2)^34^, which cooperatively induce Fas ligand (FasL/CD95L) expression in endothelial cells. FasL is a death mediator detected in solid tumor vasculature, but not in normal vasculature. It is associated with predominance of FoxP3 + Tregs and scarce CD8 T-cell infiltration. FasL-expressing endothelial cells acquire the ability to kill CD8 T-cells but not Tregs, which express higher levels of cFLIP (anti-apoptotic regulator)^35^. Tumor angiogenesis by VEGF also promotes expansion of Tregs and myeloid-derived suppressor cells (MDSC), although there was no significant difference seen in MDSC infiltration in our study. Accordingly, expression of the four cytokine genes (*IL10, VEGFA, PTGER2, FASLG*) was higher in the EAM overexpression group (EAM+) compared to the remaining group (EAM-). In lung adenocarcinoma, 16.4% (43 out of 262 patients) and 15.2% (39 out of 256 patients) showed cytokine gene overexpression in EAM+ and EAM-, respectively. In lung SCC, 18.7% (40 out of 214) and 12.2% (35 out of 288) showed cytokine gene overexpression in EAM+ and EAM− respectively.

On the contrary, activated B-cells and macrophages, among other immune cells, were significantly elevated within EAM overexpressing tumors. Recently, B-cells were shown to attenuate antitumor immunity^36,37^ and autoimmune disease^38^. Another study demonstrated an inverse correlation between activated B-cells and CD8 T-cells, while also showing a positive correlation between B-cells and Tregs^39^. This aligns with our result of lower infiltration of CD4 and CD8 T-cells and higher infiltration of B-cells and Tregs in the EAM overexpression group. Tumor associated macrophages (TAMs) have become important therapeutic targets due to their significant role in the TME. Classically activated macrophages (M1) decrease tumor cell viability and growth, whereas alternatively activated macrophages (M2) induce angiogenesis and produce survival factors of tumor cells (TGF-β, EGF, IL-6, IL-8)^40^. TAMs can also express PD-L1 upon activation of hypoxia-inducible factor-1α in hypoxic tumor regions, further inhibiting CD8 T-cell activation^41^. Recruitment of TAMs, B-cells, and Tregs by EAM may cause inactivation of the already decreased number of intra-tumoral CD8 T-cells. This suggests that EAM status may serve as a possible marker of immune exclusion, or resistance to T-cell-mediated immunotherapy.

Further, overexpression of adhesion molecules have been associated with increased metastasis. Tumor induced cytokines, such as IL-1, TNFα, and INFγ, can directly activate endothelial cells to express E/P-selectin, VCAM, and ICAM2, thereby triggering activation of integrins^42,43^. A previous study highlighted that adhesion of colon cancer cells to endothelial cells expressing E-selectin activates the ERK/MAPK (extracellular signal-regulated kinase/mitogen-activated protein kinase) pathway in endothelial cells. This results in the dissociation of VE-cadherin/β-catenin complex and the loss of adherens junction, leading to increased permeability and extravasation of cancer cells^44^. We also evaluated the association between cancer stage/metastasis and EAM expression, but further investigation was difficult due to the uneven distribution of patient samples regarding cancer stage. Only 4.9% and 1.4% of samples were advanced stage patients (stage IV) in lung adenocarcinoma and SCC respectively. Additional studies with greater statistical power may help to clarify the correlation between metastasis and EAM expression.

We also observed differential infiltration of immune cells in patient samples that overexpressed CBM genes, specifically in desmosomes (Fig. 3). Infiltration of activated CD4, CD8, effector memory CD4 T-cells, and Th17 cells were higher in the desmosome overexpression group, whereas infiltration of mast cells, macrophages, Tregs, and activated B-cells were lower in lung adenocarcinoma. In lung SCC, only Th17 cells were significantly higher in desmosome gene overexpressing samples. In order to reveal the association between EAM and CBM expression, we compared genes encoding seven desmosomal proteins (DST, DSC3, JUP, DSP, PPL, PKP3, DSG1) and seven adhesion molecules (ITGA4, ITGB1, ITGB7, JAM3, ICAM2, SELE, SELL). Both sets of genes were highly expressed in lung adenocarcinoma and SCC in a mutually exclusive manner (Fig. 4), implying that EAM and CBM expression are independent. Overall survival was not significantly different between the three CBM overexpression groups in both lung adenocarcinoma and SCC (Figure S8).

In contrast to a previous study which reported that overexpression of mechanical barrier molecule genes (DST, DSC3, DSP, PPL, PKP3, JUP, FLG, and TACSTD2) correlated with decreased CD8 T-cell infiltration in human melanoma and ovarian carcinoma^10^, our study demonstrates that overexpression of desmosome genes correlates with higher CD8 T-cell infiltration in lung adenocarcinoma. Additionally, elevated level of DSC3 was reported to increase metastatic risk in melanoma, whereas DSC3 was associated with better prognosis in lung and colon cancer^11–13^. This suggests that the same barrier molecule may have differential effects in the TME specific for the cancer histology. Additionally, elevated expression of desmoglein3 (DSG3) and plakoglobin (JUP) have been linked to lower tumor grade and improved clinical outcome in NSCLC^45,46^. However, we found that the overexpression of CBM genes did not predict better survival outcomes in lung adenocarcinoma and SCC. Further validation of patient survivability in larger datasets including advanced stage patient samples is needed to better demonstrate the clinical relevance of CBM expression status in current immunotherapy.

We also analyzed CD8 T-cell score generated by four signature genes (CD8A, CD8B, IFNG, and PRF1). In the EAM overexpression group, CD8 T-cell score was high whereas activated CD4 and CD8 T-cell infiltration was low in both types of lung cancers (Fig. 5A,B). On the contrary, CD8 T-cell score was low in the desmosome overexpression group in lung SCC (Fig. 5C). CD8 T-cell score may not directly reflect both activated and inactivated CD8 T-cells. As a result, CD8 T-cell score can be high even though the actual activated CD8 T-cell infiltration is low. One possible mechanism for this finding can be explained by the previously mentioned T-cell inactivation by Treg and B-cells. In the EAM overexpression group, the selectively increased intra-tumoral Tregs and B-cells may inactivate CD4 and CD8 T-cells, causing low infiltration of activated CD8 T-cells despite high CD8 T-cell score. Similarly in the desmosome overexpression group, activated CD8 T-cell infiltration was not lower in spite of the low CD8 T-cell score. This can also be explained by the lack of T-cell inactivation by Tregs and B cells.

In addition, we evaluated relationships between EAM/CBM gene expression and the β-catenin/TGF-β/INF-γ pathways, which have been implicated in CD8 T-cell exclusion, apoptosis, and recognition, respectively. Interestingly, expression of these immune exclusion signatures was found to be independent of expression of EAM/CBM genes (Figure S6). In β-catenin gene pairs, only 2 (with EAM genes in adenocarcinoma), 3 (with EAM genes in SCC), 4 (with CBM genes in adenocarcinoma), and 1 (with CBM genes in SCC) out of 42 different gene pairs significantly converged towards co-occurrence. Additionally, in TGF-β gene pairs, only 6 (with EAM genes in adenocarcinoma), 5 (with EAM genes in SCC), 2 (with CBM genes in adenocarcinoma), and 5 (with CBM genes in SCC) out of 42 different gene pairs significantly co-occurred. In INF-γ, 8 (with EAM genes in adenocarcinoma), 15 (with EAM genes in SCC), 1 (with CBM genes in adenocarcinoma), and 0 (with CBM genes in SCC) out of 42 different gene pairs significantly converged towards co-occurrence (Table S2,S3). Additional studies may be necessary to examine the role of these gene signatures in T-cell exclusion and to define what subsets of these pathways may be most valuable as prognostic biomarkers.

In summary, we demonstrate for the first time that overexpression of EAM and desmosome genes are linked to differential infiltration of various immune cells in human lung cancer tissue. A limitation of the present study is that our analysis was purely *in silico*, and thus the clinical relevance of our results has not yet been examined. Further investigation is required to better understand the system governing adhesion and barrier molecules and their immune modulatory effect in the TME. The present finding of the correlation between selective immune cell infiltration with EAM and desmosome expression may represent under-appreciated mechanisms underlying T-cell infiltration in lung tumors. Our data suggest that EAM and desmosome status can be used as a potential biomarker for subsets of tumors that fail to respond to current immunotherapies.

## Electronic supplementary material

Supplementary figures
