# Supplementary material for: Overexpression of adhesion molecules and barrier molecules is associated with differential infiltration of immune cells in non-small cell lung cancer
Source: Sci Rep. 2018 Jan 18;8:1023. doi: 10.1038/s41598-018-19454-3 (PMC5773521; doi:10.1038/s41598-018-19454-3)
Supplement: Supplementary file 1 — Supplementary figures [file 41598_2018_19454_MOESM1_ESM.pdf]

Overexpression of adhesion molecules and barrier molecules is associated with differential infiltration of immune cells in non-small cell lung cancer

Young Kwang Chae[1,2,+], Wooyoung M. Choi[2,+], William H. Bae[2], Jonathan Anker[2], Andrew A. Davis[2], Sarita Agte[1], Wade T. Iams[2], Marcelo Cruz[2], Maria Matsangou[1,2], Francis J. Giles[1,2]

[1]Robert H. Lurie Comprehensive Cancer Center of Northwestern University, Chicago, 60611, USA

[2]Northwestern University Feinberg School of Medicine, Chicago, 60611, USA

Corresponding author:

Co-Director, Early Phase Clinical Trials Unit

Developmental Therapeutics - Lurie Cancer Center

Assistant Professor, Department of Medicine

Northwestern University Feinberg School of Medicine

Robert H. Lurie Comprehensive Cancer Center of Northwestern University

645 N. Michigan Avenue | Suite 1006 | Chicago, IL 60611

Tel: 312 926 4248 | Fax: 312 695 0370

Email: young.chae@northwestern.edu

[+]these authors contributed equally to this work as first author.

A.

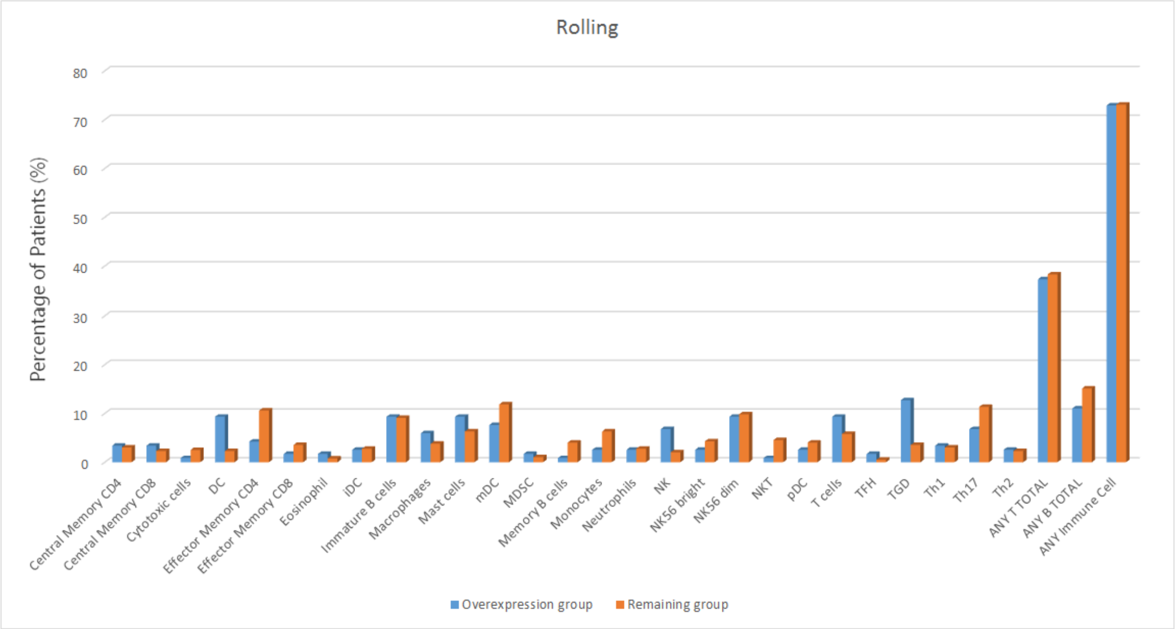

B.

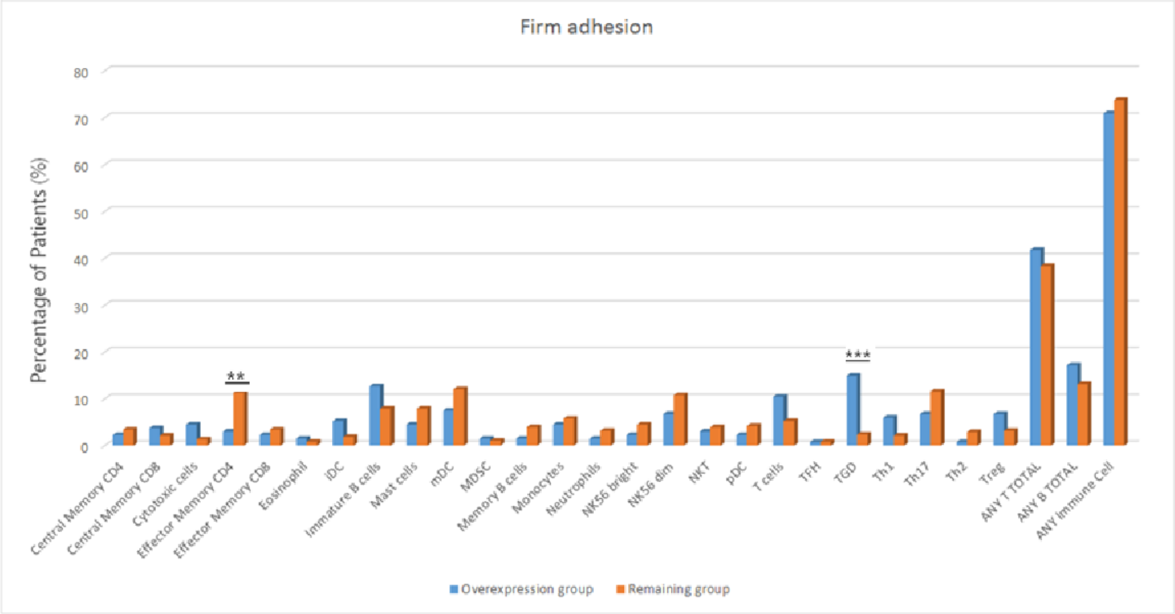

C.

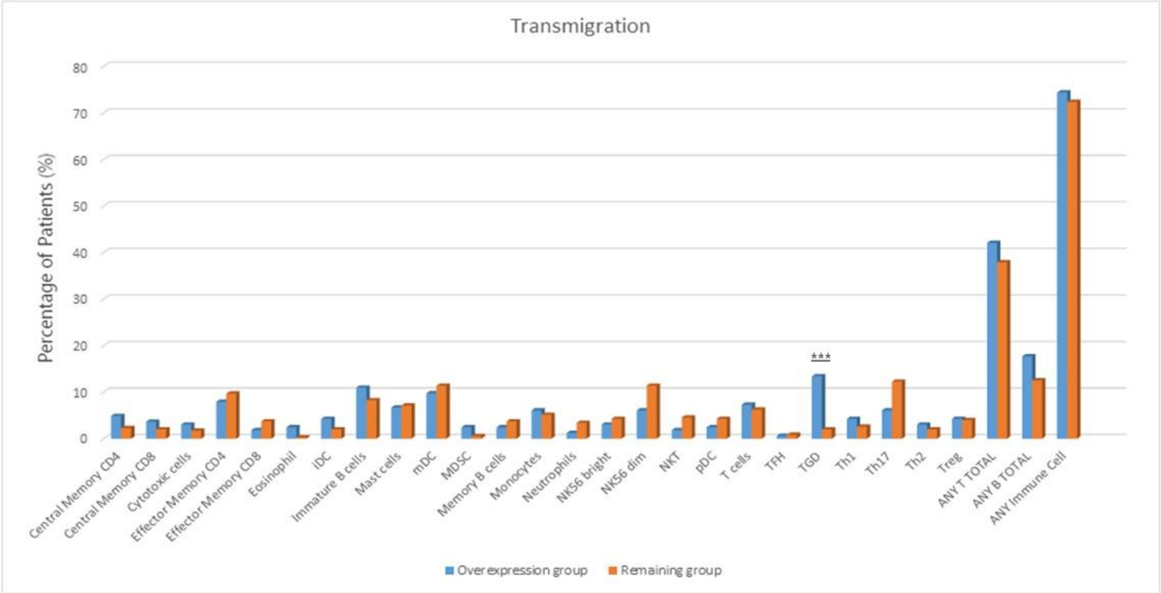

Figure S1. Overall immune cell infiltration landscape by endothelial adhesion molecule (EAM) status in lung adenocarcinoma. A. Rolling gene overexpression group compared to remaining group. **B.** Firm adhesion gene overexpression group compared to remaining group. **C.** Transmigration gene overexpression group compared to remaining group. ** p<0.05, ** p<0.01, *** p<0.001.*

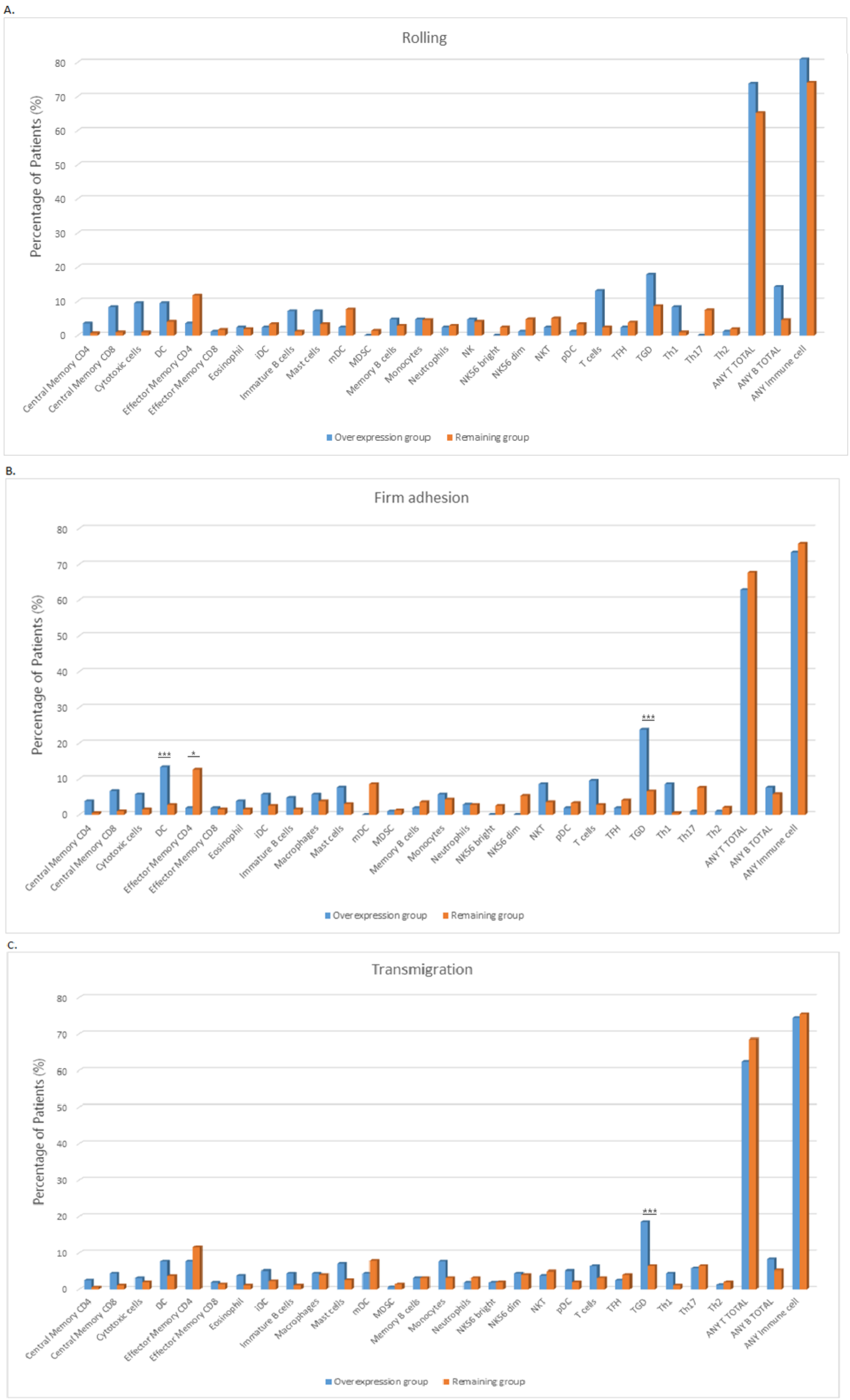

Figure S2. Overall immune cell infiltration landscape by EAM status in lung squamous cell carcinoma (SCC). A. Rolling gene overexpression group compared to remaining group. **B.** Firm adhesion gene overexpression group compared to remaining group. **C.** Transmigration gene overexpression group compared to remaining group. ** p<0.05, ** p<0.01, *** p<0.001.*

A.

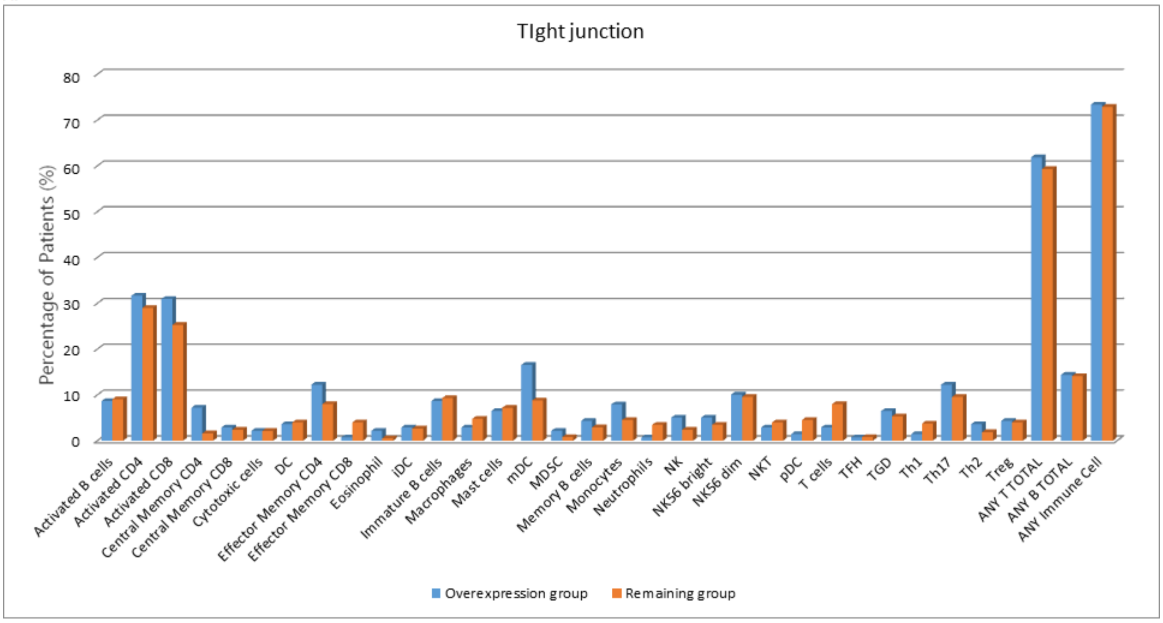

B.

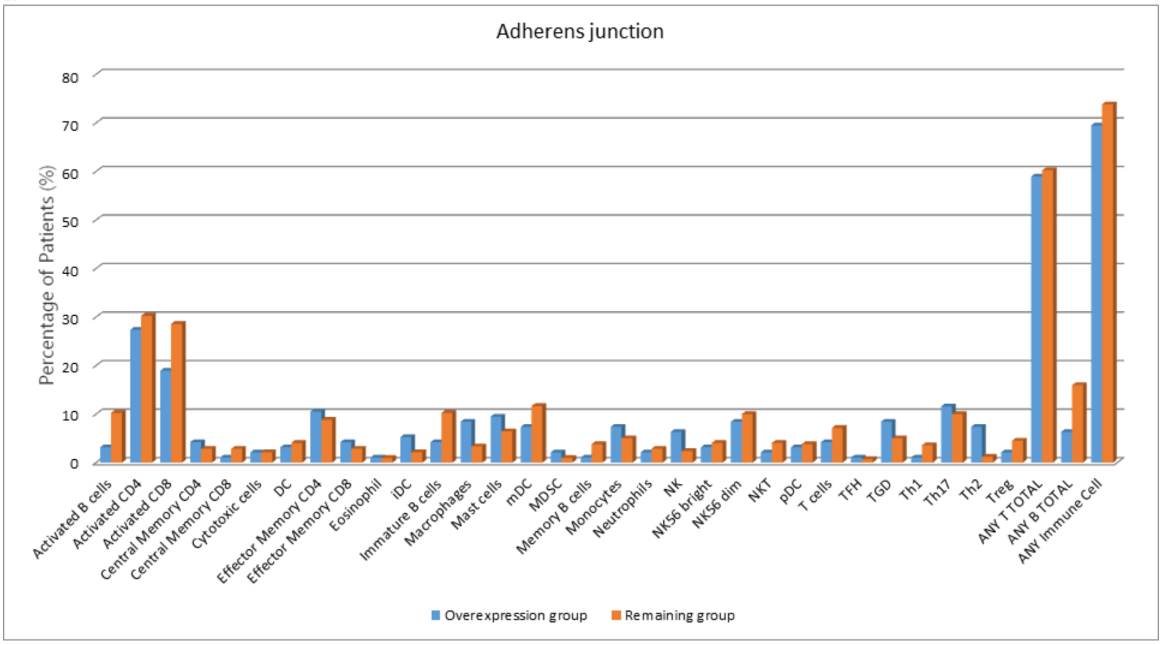

C.

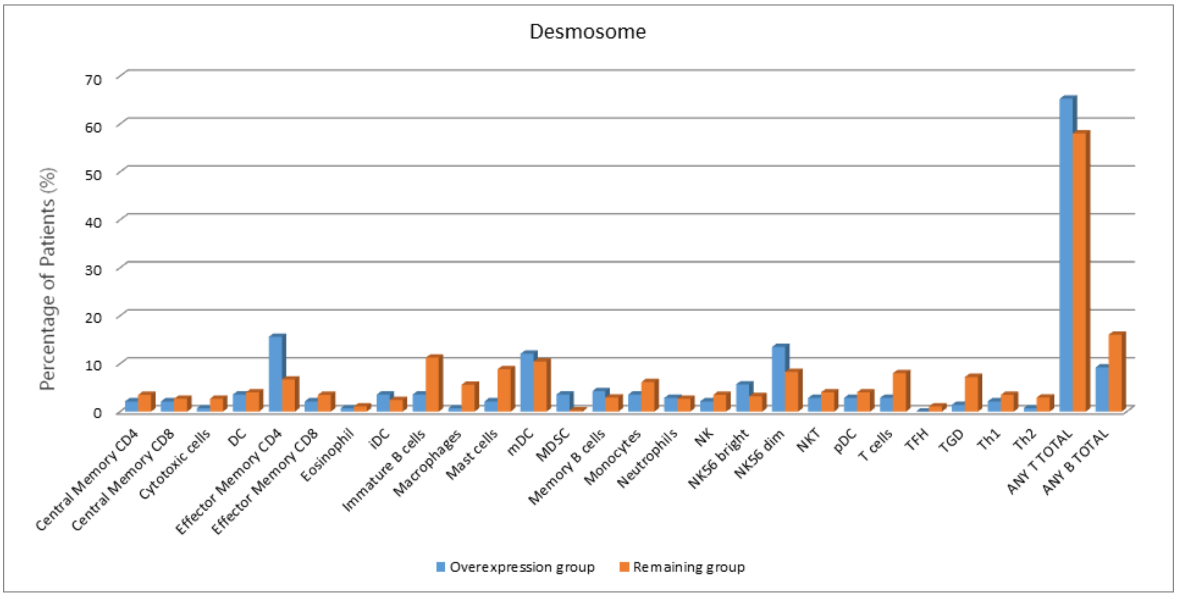

Figure S3. Overall immune cell infiltration landscape by cellular barrier molecule (CBM) status in lung adenocarcinoma. A. Tight junction gene overexpression group compared to remaining group. **B.** Adherens junction gene overexpression group compared to remaining group. **C.** Desmosome gene overexpression group compared to remaining group. *p values>0.05*

A.

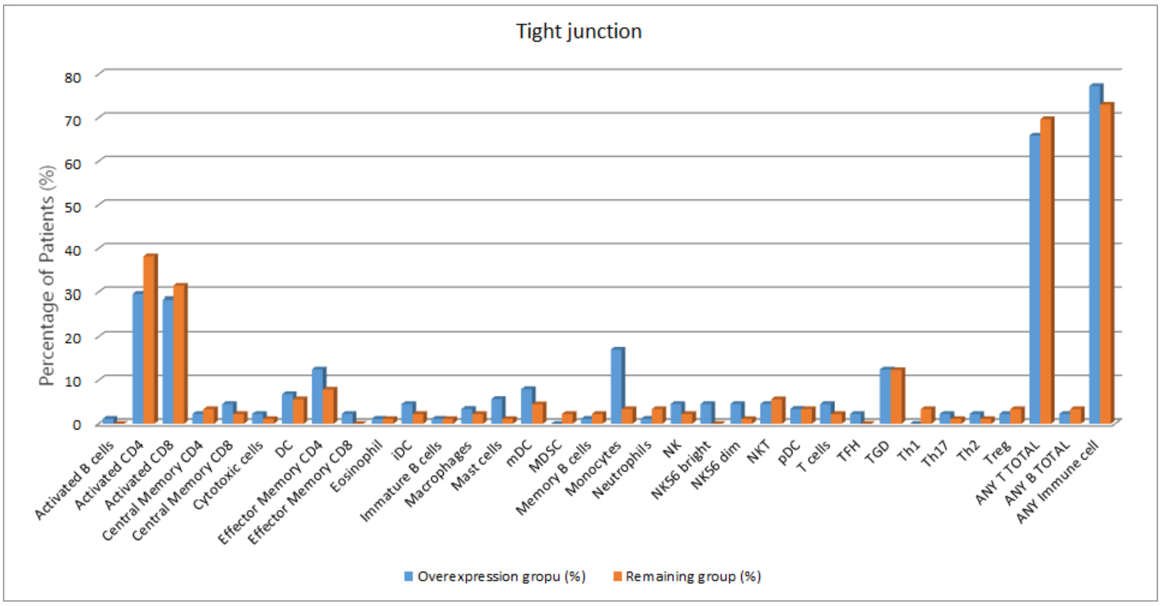

B.

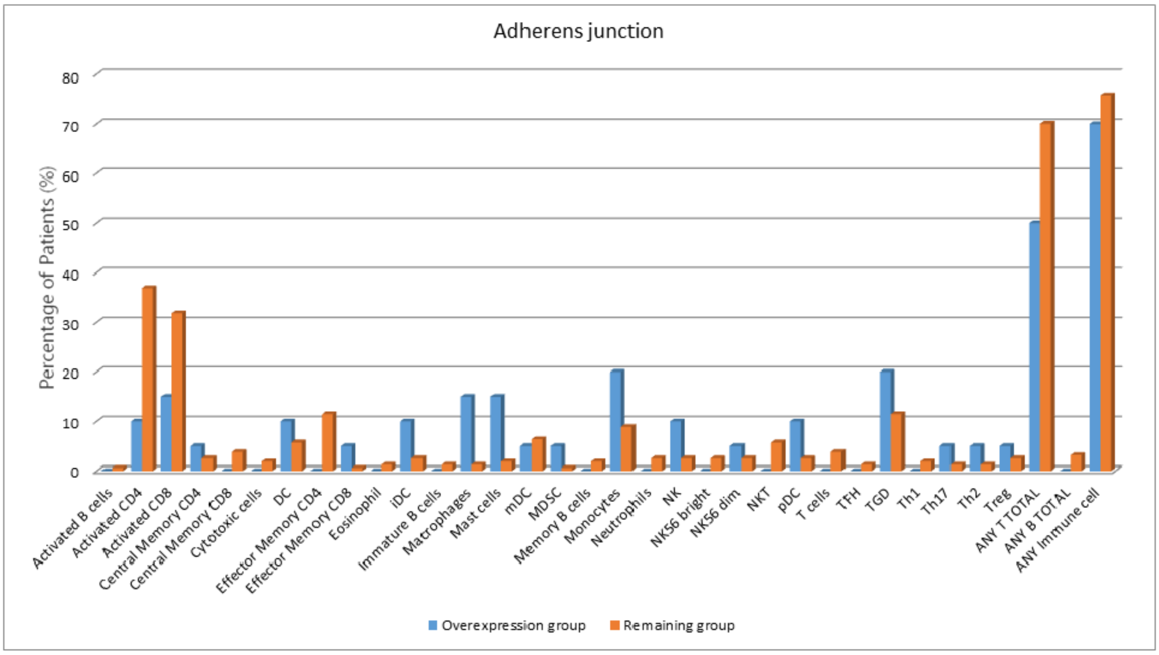

C.

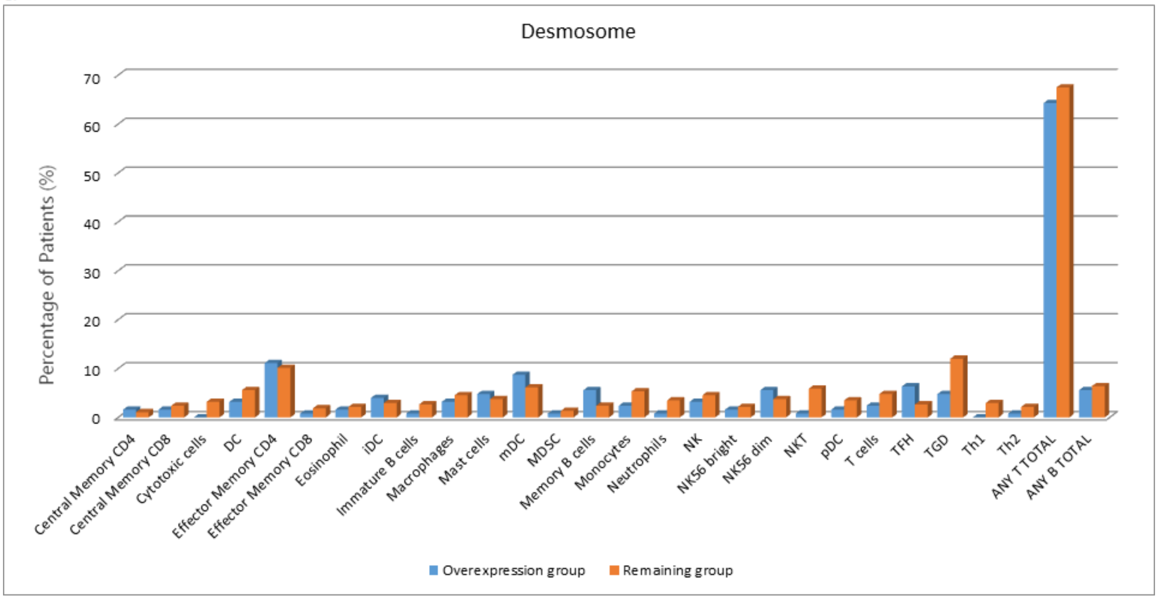

Figure S4. Overall immune cell infiltration landscape by CBM status in lung SCC. A. Tight junction gene overexpression group compared to remaining group. **B.** Adherens junction gene overexpression group compared to remaining group. **C.** Desmosome gene overexpression group compared to remaining group. *p values>0.05.*

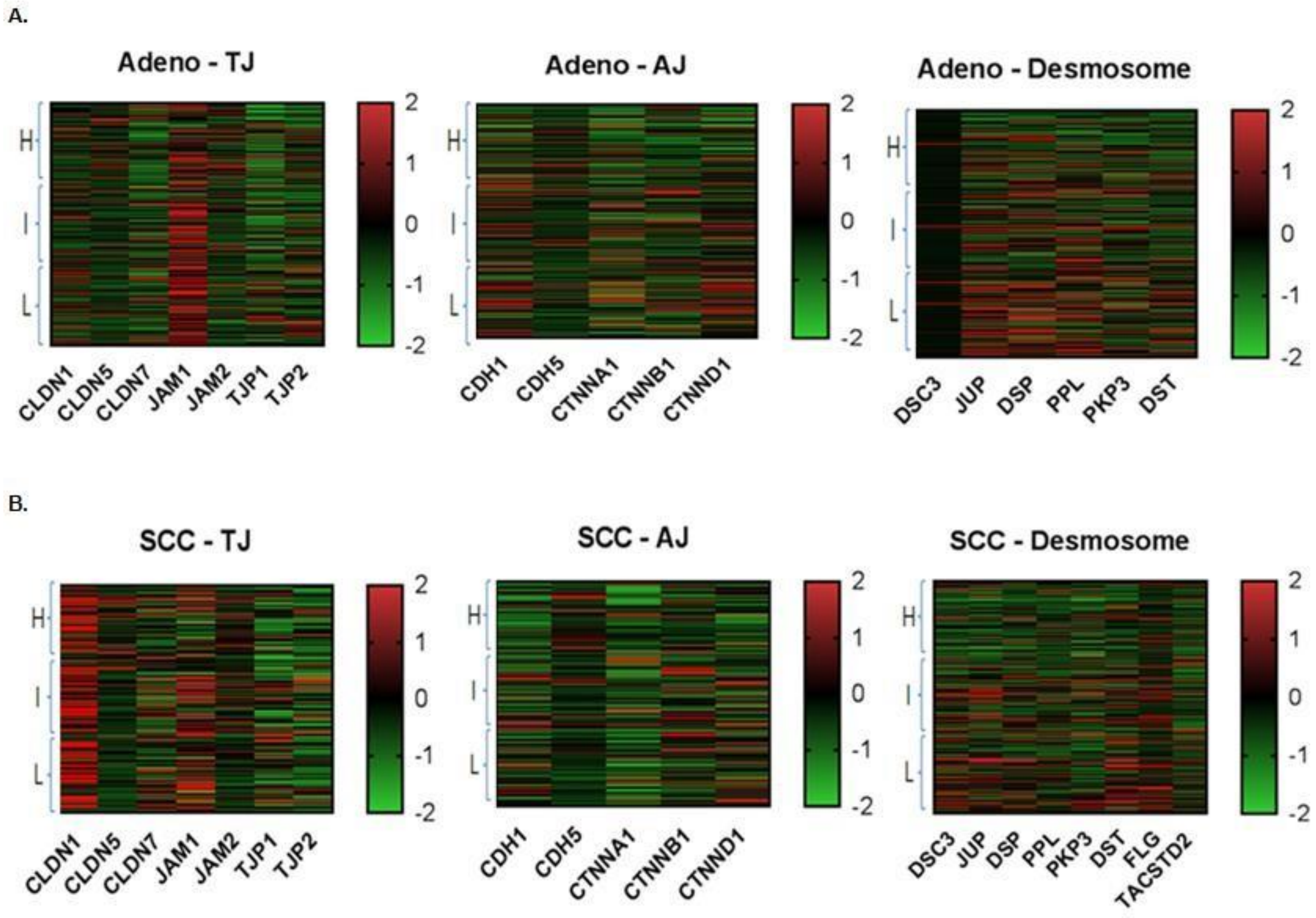

Figure S5. Heat maps of CD8 T cell signature gene expression in each CBM gene status.
A. Lung adenocarcinoma. **B.** Lung SCC.
Right column bars indicate mRNA-seq z scores. Abbreviations: TJ, tight junction: AJ, adherens junction: H, high: I, intermediate: L, low expression of CD8 T cell signature genes.

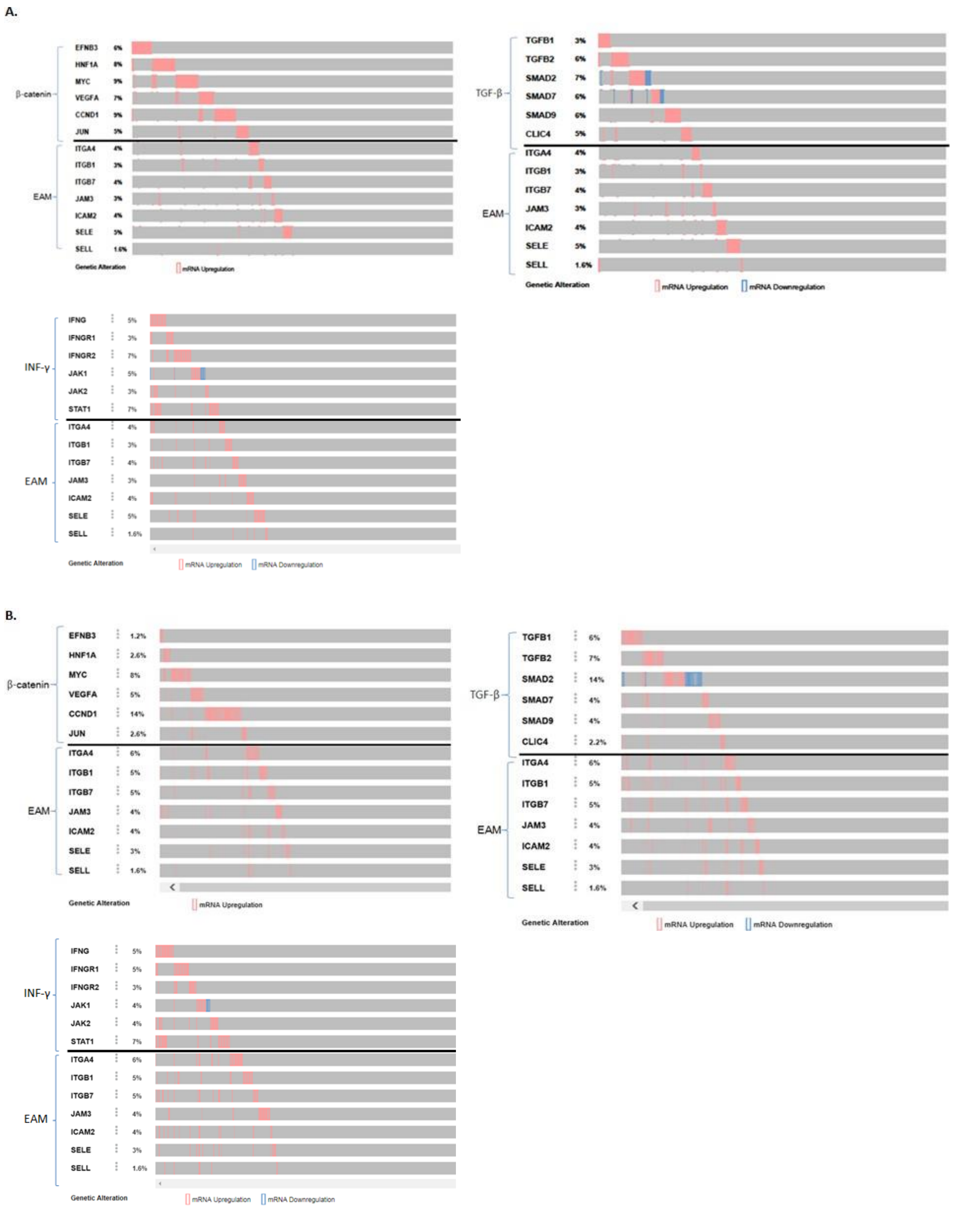

Figure S6. OncoPrint of β-catenin/TGF-β/INF-γ pathway related gene expression with association to EAM gene expression.
A. Lung adenocarcinoma. **B.** Lung SCC. The distribution pattern of each gene sets is independent from each other.

A.

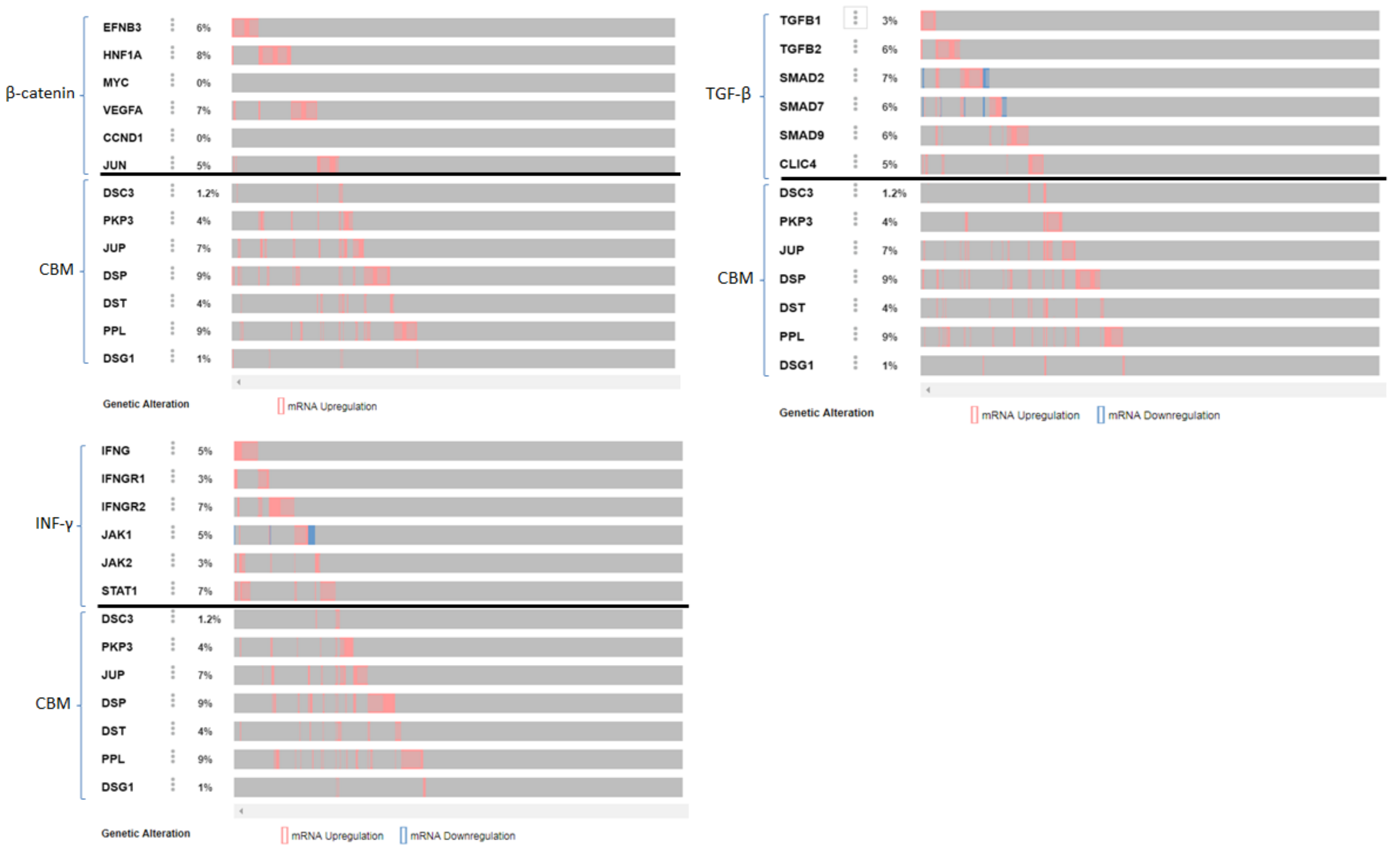

B.

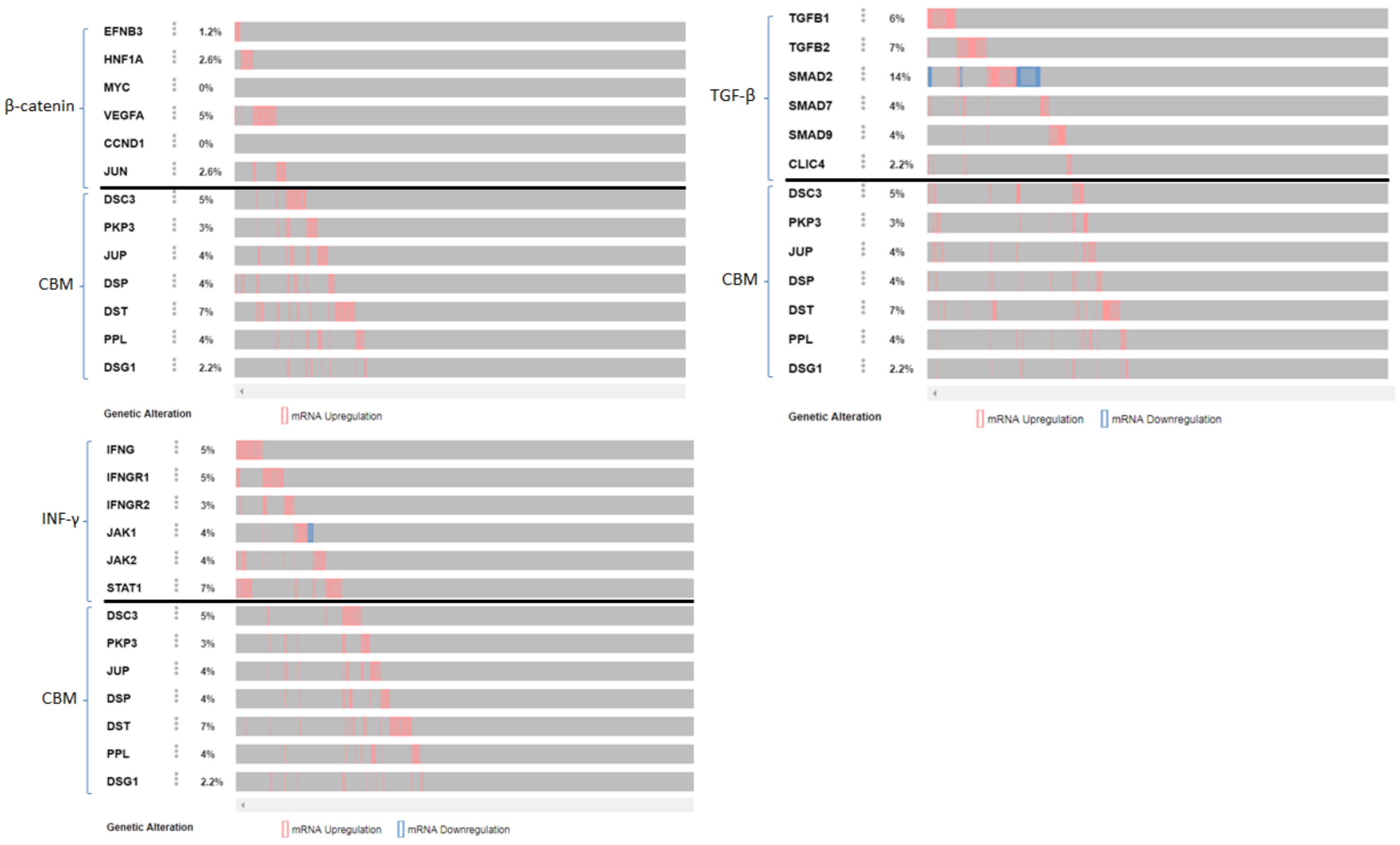

Figure S7. OncoPrint of β-catenin/TGF-β/INF-γ pathway related gene expression with association to CBM gene expression.
A. Lung adenocarcinoma. **B.** Lung SCC. The distribution pattern of each gene sets is independent from each other.

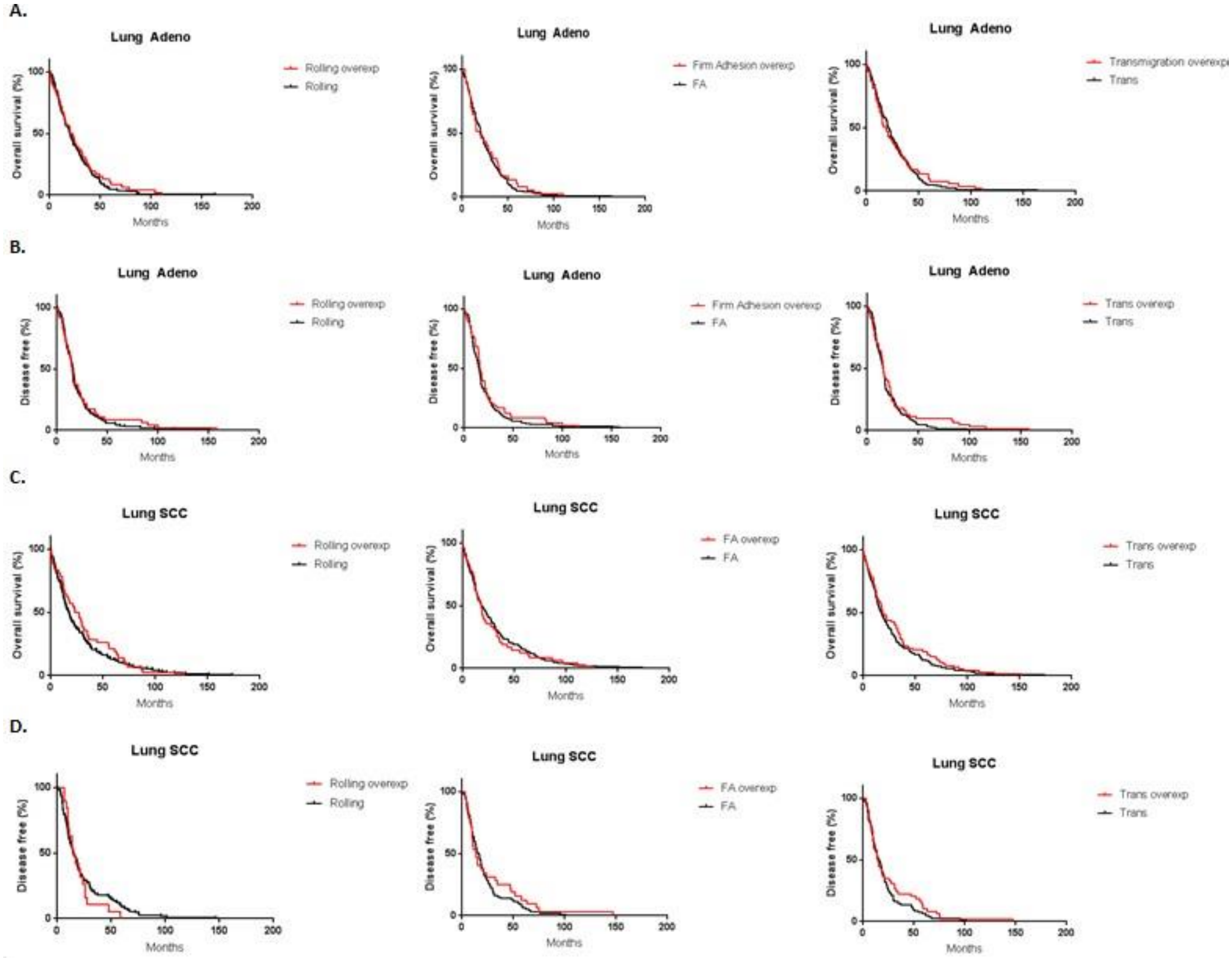

Figure S8. Overall survival and disease free survival in EAM overexpression group.
A. Overall survival (%) in lung adenocarcinoma. **B.** Disease free survival (%) in lung adenocarcinoma. **C.** Overall survival (%) in lung SCC. **D.** Disease free survival (%) in lung SCC.
Red line, overexpression group: Black line, remaining group. Abbreviations: FA, firm adhesion: Trans, transmigration.

A.

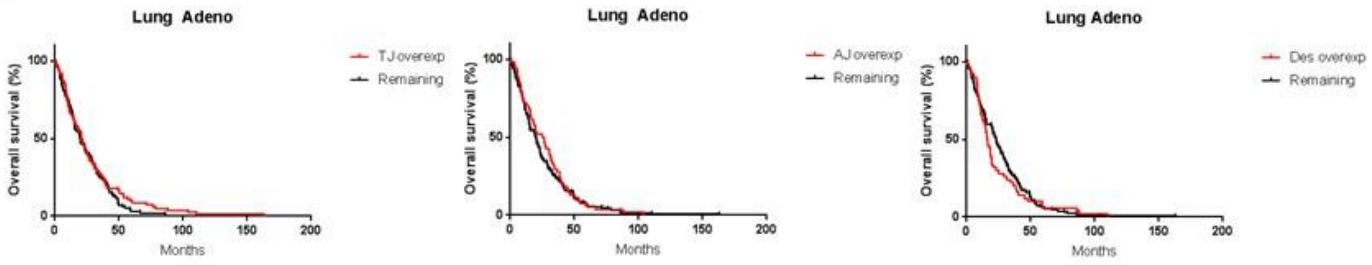

B.

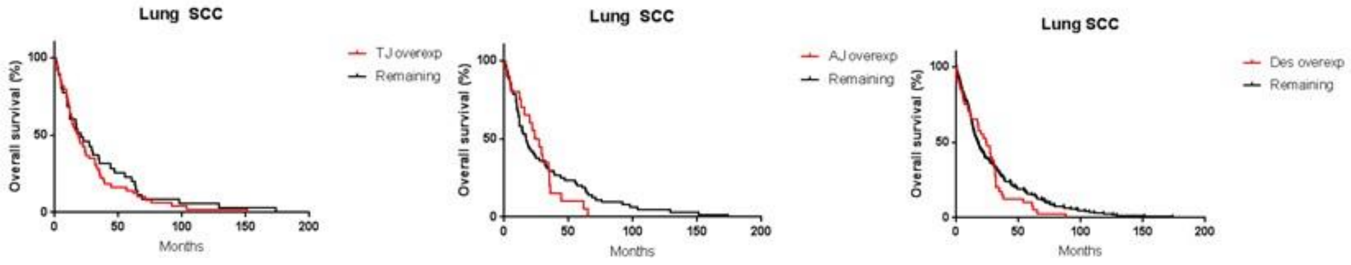

Figure S9. Overall survival in CBM overexpression group.
A. Overall survival (%) in lung adenocarcinoma. **B.** Overall survival (%) in lung SCC.
Red line, overexpression group: Black line, remaining group. Abbreviations: TJ, tight junction: AJ, adherens junction: Des, desmosome.

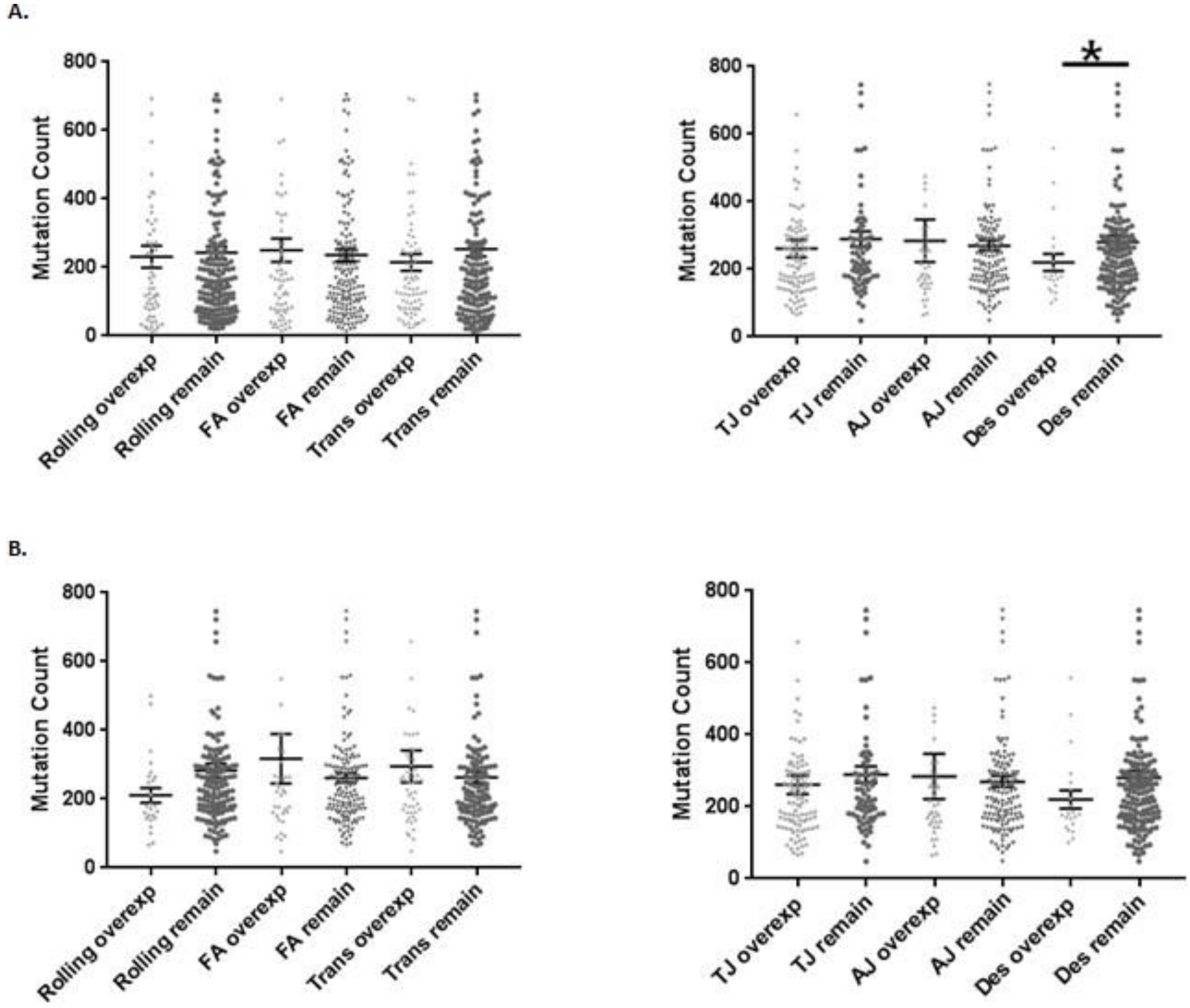

Figure S10: Comparison of tumor mutation burden between EAM/CBM overexpression group and remaining group.
A. Lung adenocarcinoma. **B.** Lung SCC. *Abbreviations: FA, firm adhesion: Trans, transmigration: TJ, tight junction: AJ, adherens junction: Des, desmosome. * p<0.05.*

A.

Gene A	Gene B	p value	Log Odds Ratio	Association
DST	DSC3	<0.001	>3	Tendency towards co-occurrence
JUP	PKP3	<0.001	2.628	Tendency towards co-occurrence
ITGA4	ITGB8	<0.001	2.313	Tendency towards co-occurrence
DSC3	DSC1	0.001	>3	Tendency towards co-occurrence
DST	DSP	0.005	1.612	Tendency towards co-occurrence
DSC3	JUP	0.005	2.673	Tendency towards co-occurrence
DST	PKP3	0.007	1.906	Tendency towards co-occurrence
ITGB1	JAM3	0.018	1.936	Tendency towards co-occurrence
DST	PPL	0.02	1.381	Tendency towards co-occurrence
**DST**	**ITGB1**	**0.021**	**1.869**	**Tendency towards co-occurrence**
DSC3	PKP3	0.025	2.457	Tendency towards co-occurrence
DST	JUP	0.036	1.357	Tendency towards co-occurrence
JUP	DSP	0.039	0.952	Tendency towards co-occurrence
ITGB7	SELL	0.041	2.098	Tendency towards co-occurrence
SELE	SELL	0.057	1.907	Tendency towards co-occurrence
DSP	ITGB1	0.063	1.16	Tendency towards co-occurrence

B.

Gene A	Gene B	p value	Log Odds Ratio	Association
DSC3	DSP	<0.001	2.618	Tendency towards co-occurrence
DSP	PPL	<0.001	>3	Tendency towards co-occurrence
DSP	DSG1	<0.001	2.967	Tendency towards co-occurrence
ITGA4	ITGB7	<0.001	2.191	Tendency towards co-occurrence
ITGA4	ICAM2	<0.001	2.267	Tendency towards co-occurrence
ITGA4	SELL	<0.001	>3	Tendency towards co-occurrence
ITGB7	ICAM2	<0.001	>3	Tendency towards co-occurrence
ITGB7	SELE	<0.001	2.322	Tendency towards co-occurrence
ITGB7	SELL	<0.001	>3	Tendency towards co-occurrence
ICAM2	SELE	<0.001	>3	Tendency towards co-occurrence
ICAM2	SELL	<0.001	>3	Tendency towards co-occurrence
DSC3	JUP	0.002	2.082	Tendency towards co-occurrence
DSP	PKP3	0.019	1.902	Tendency towards co-occurrence
SELE	SELL	0.027	2.363	Tendency towards co-occurrence
PPL	DSG1	0.061	1.822	Tendency towards co-occurrence
DST	DSC3	0.062	1.145	Tendency towards co-occurrence

Table S1. Mutual exclusivity of EAM genes and desmosome genes.
A. Lung adenocarcinoma. Genes that showed tendency towards co-occurrence were either in the same group of EAM genes or desmosome genes except for EAM gene *ITGB1* and desmosome gene *DST*. **B.** Lung SCC. All genes that showed tendency towards co-occurrence were either in the same group of EAM genes or desmosome genes.

A. Lung adenocarcinoma

β-catenin	EAM	p value	Log Odds Ratio	Association
**HNF1A**	**JAM3**	**0.043**	**1.301**	**Tendency towards co-occurrence**
**JUN**	**SELE**	**0.045**	**1.264**	**Tendency towards co-occurrence**

Lung SCC

β-catenin	EAM	p value	Log Odds Ratio	Association
**HNF1A**	**JAM3**	**0.002**	**2.458**	**Tendency towards co-occurrence**
**EFNB3**	**JAM3**	**0.001**	**>3**	**Tendency towards co-occurrence**
**CCND1**	**ITGB7**	**0.027**	**<-3**	**Tendency towards co-occurrence**

B. Lung adenocarcinoma

TGF-β	EAM	p value	Log Odds Ratio	Association
**SMAD9**	**JAM3**	**<0.001**	**2.249**	**Tendency towards co-occurrence**
**CLIC4**	**ITGB1**	**<0.001**	**2.252**	**Tendency towards co-occurrence**
**TGFB2**	**ITGB1**	**0.002**	**2.069**	**Tendency towards co-occurrence**
**SMAD2**	**ITGB1**	**0.005**	**1.774**	**Tendency towards co-occurrence**
**CLIC4**	**ITGA4**	**0.012**	**1.753**	**Tendency towards co-occurrence**
**TGFB1**	**ITGB1**	**0.015**	**2.007**	**Tendency towards co-occurrence**

Lung SCC

TGF-β	EAM	p value	Log Odds Ratio	Association
**SMAD7**	**SELE**	**<0.001**	**2.721**	**Tendency towards co-occurrence**
**SMAD9**	**JAM3**	**<0.001**	**2.215**	**Tendency towards co-occurrence**
**SMAD7**	**ICAM2**	**0.003**	**2.194**	**Tendency towards co-occurrence**
**SMAD7**	**ITGB1**	**0.010**	**1.796**	**Tendency towards co-occurrence**
**TGFB2**	**ITGB1**	**0.021**	**1.342**	**Tendency towards co-occurrence**

C. Lung adenocarcinoma

INF-γ	EAM	p value	Log Odds Ratio	Association
**IFNG**	**ITGA4**	**0.002**	**2.045**	**Tendency towards co-occurrence**
**IFNG**	**ITGB7**	**0.004**	**1.844**	**Tendency towards co-occurrence**
**IFNG**	**ICAM2**	**0.019**	**1.577**	**Tendency towards co-occurrence**
**IFNGR1**	**ICAM2**	**0.027**	**1.747**	**Tendency towards co-occurrence**
**JAK1**	**ITGB7**	**0.026**	**1.473**	**Tendency towards co-occurrence**
**JAK2**	**ITGA4**	**<0.001**	**2.672**	**Tendency towards co-occurrence**
**JAK2**	**ITGB7**	**0.004**	**2.109**	**Tendency towards co-occurrence**
**STAT1**	**ITGA4**	**0.036**	**1.357**	**Tendency towards co-occurrence**

Lung SCC

INF-γ	EAM	p value	Log Odds Ratio	Association
**STAT1**	**ITGB7**	**<0.001**	**2.044**	**Tendency towards co-occurrence**
**JAK1**	**SELL**	**<0.001**	**>3**	**Tendency towards co-occurrence**
**IFNG**	**ITGB7**	**<0.001**	**1.979**	**Tendency towards co-occurrence**
**STAT1**	**ICAM2**	**0.001**	**1.904**	**Tendency towards co-occurrence**
**JAK1**	**ITGB7**	**0.002**	**2.026**	**Tendency towards co-occurrence**
**IFNG**	**ICAM2**	**0.002**	**2.011**	**Tendency towards co-occurrence**
**JAK1**	**SELE**	**0.004**	**2.134**	**Tendency towards co-occurrence**
**JAK1**	**ITGA4**	**0.004**	**1.826**	**Tendency towards co-occurrence**
**JAK2**	**ITGA4**	**0.005**	**1.763**	**Tendency towards co-occurrence**
**JAK1**	**ICAM2**	**0.006**	**1.987**	**Tendency towards co-occurrence**
**JAK2**	**ICAM2**	**0.007**	**1.928**	**Tendency towards co-occurrence**

**IFNGR1**	**ITGB1**	**0.008**	**1.638**	**Tendency towards co-occurrence**
**JAK2**	**ITGB7**	**0.017**	**1.629**	**Tendency towards co-occurrence**
**JAK2**	**SELE**	**0.034**	**1.657**	**Tendency towards co-occurrence**
**JAK2**	**SELL**	**0.044**	**2.065**	**Tendency towards co-occurrence**

Table S2. Co-occurrence of β-catenin/TGF-β/ INF- γ genes and EAM genes.

Gene pairs showing statistically significant (p value < 0.05) tendency towards co-occurrence were selected.

A. Co-occurrence between β-catenin genes and EAM genes in lung adenocarcinoma and SCC. **B.** Co-occurrence between TGF-β genes and EAM genes in lung adenocarcinoma and SCC. **C.** Co-occurrence between INF-γ genes and EAM genes in lung adenocarcinoma and SCC.

A. Lung adenocarcinoma

β-catenin	CBM	p value	Log Odds Ratio	Association
**HNF1A**	**PKP3**	**0.006**	**1.563**	**Tendency towards co-occurrence**
**VEGFA**	**DSP**	**0.01**	**1.186**	**Tendency towards co-occurrence**
**JUN**	**DST**	**0.015**	**1.661**	**Tendency towards co-occurrence**
**HNF1A**	**JUP**	**0.05**	**0.967**	**Tendency towards co-occurrence**

Lung SCC

β-catenin	CBM	p value	Log Odds Ratio	Association
**VEGFA**	**DST**	**0.005**	**1.566**	**Tendency towards co-occurrence**

B. Lung adenocarcinoma

TGF-β	CBM	p value	Log Odds Ratio	Association
**TGFB2**	**PPL**	**0.003**	**1.458**	**Tendency towards co-occurrence**
**CLIC4**	**DSC3**	**0.032**	**2.317**	**Tendency towards co-occurrence**

Lung SCC

TGF-β	CBM	p value	Log Odds Ratio	Association
**TGFB1**	**JUP**	**<0.001**	**2.208**	**Tendency towards co-occurrence**
**TGFB1**	**PKP3**	**<0.001**	**2.263**	**Tendency towards co-occurrence**
**TGFB1**	**DSC3**	**0.011**	**1.535**	**Tendency towards co-occurrence**
**SMAD2**	**DSC3**	**0.027**	**1.077**	**Tendency towards co-occurrence**
**CLIC4**	**PKP3**	**0.05**	**1.951**	**Tendency towards co-occurrence**

C. Lung adenocarcinoma

INF-γ	CBM	p value	Log Odds Ratio	Association
**JAK1**	**DSP**	**0.036**	**1.066**	**Tendency towards co-occurrence**

Lung SCC

INF-γ	CBM	p value	Log Odds Ratio	Association
**No tendency towards co-occurrence**				

Table S3. Co-occurrence of β-catenin/TGF-β/ INF- γ genes and CBM genes.

Gene pairs showing statistically significant (p value < 0.05) tendency towards co-occurrence were selected.

A. Co-occurrence between β-catenin genes and CBM genes in lung adenocarcinoma and SCC. **B.** Co-occurrence between TGF-β genes and CBM genes in lung adenocarcinoma and SCC. **C.** Co-occurrence between INF- γ genes and CBM genes in lung adenocarcinoma and SCC.
